# Charge stabilization *via* electron exchange: excited charge separation in symmetric, central triphenylamine derived, dimethylaminophenyl–tetracyanobutadiene donor–acceptor conjugates†

**DOI:** 10.1039/d0sc04648e

**Published:** 2020-11-13

**Authors:** Indresh S. Yadav, Ajyal Z. Alsaleh, Rajneesh Misra, Francis D'Souza

**Affiliations:** Department of Chemistry, Indian Institute of Technology Indore 453552 India rajneeshmisra@iiti.ac.in; Department of Chemistry, University of North Texas 1155 Union Circle, #305070 Denton TX 76203-5017 USA Francis.DSouza@UNT.edu

## Abstract

Photoinduced charge separation in donor–acceptor conjugates plays a pivotal role in technology breakthroughs, especially in the areas of efficient conversion of solar energy into electrical energy and fuels. Extending the lifetime of the charge separated species is a necessity for their practical utilization, and this is often achieved by following the mechanism of natural photosynthesis where the process of electron/hole migration occurs distantly separating the radical ion pairs. Here, we hypothesize and demonstrate a new mechanism to stabilize the charge separated states *via* the process of electron exchange among the different acceptor entities in multimodular donor–acceptor conjugates. For this, star-shaped, central triphenylamine derived, dimethylamine–tetracyanobutadiene conjugates have been newly designed and characterized. Electron exchange was witnessed upon electroreduction in conjugates having multiple numbers of electron acceptors. Using ultrafast spectroscopy, the occurrence of excited state charge separation, and the effect of electron exchange in prolonging the lifetime of charge separated states in the conjugates having multiple acceptors have been successfully demonstrated. This work constitutes the first example of stabilizing charge-separated states *via* the process of electron exchange.

## Abstract 2

The significance of electron exchange in stabilizing the charge-separated state is revealed in multi-modular donor–acceptor conjugates.

## Graphical abstract

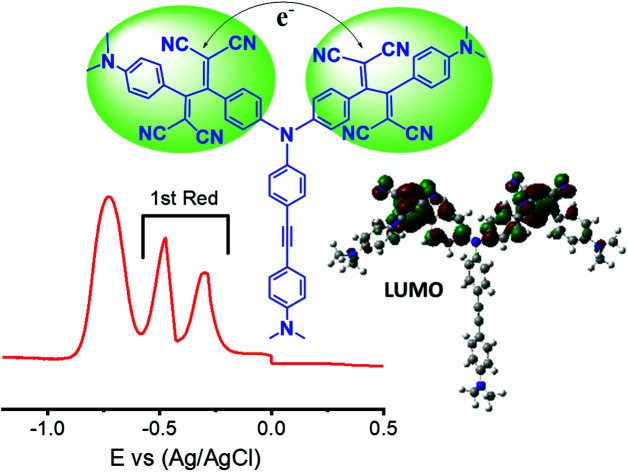

## Introduction

Excited state charge transfer in donor–acceptor conjugates is one of the widely investigated topics in recent years due to their usage in building energy harvesting photonic devices.^1–19^ Understanding the principles governing the kinetics of charge transfer and separation, securing high charge separation quantum yields, avoiding large energy losses, and prolonging the lifetime of the radical ion pairs by molecular engineering of the conjugates have been the main focus of these studies.^1–12^ In simple donor–acceptor conjugates, charge separation from the excited singlet state of the donor or acceptor can store the greatest amount of energy; however, since the process originates from the singlet excited state, the charge separated states are generally short-lived. In natural photosynthesis, the lifetime of charge separation is prolonged by subsequent electron transfer to secondary acceptors.^20–22^ This method of charge stabilization optimizes the quantum yields but comes at the cost of lowering the overall efficiency due to the energy losses encountered during secondary electron transfer steps. Alternate approaches including electron/hole delocalization in conjugates having closely interacting multiple donor or acceptor entities^23,24^ and utilization of high energy triplet sensitizers to promote charge transfer from long-lived triplet excited states to prolong the lifetime of charge separated states^25,26^ have also been proven to work.

In recent years, the design and synthesis of π-conjugated symmetrical and unsymmetrical donor–acceptor chromophores have been extensively investigated due to their potential applications in organic photovoltaics,^27–30^ molecular electronics^31^ and bioimaging.^32^ Star-shaped π-conjugated molecular systems exhibit many advantages over linear conjugated molecular systems including excellent solubility and less aggregation.^33^ Tuning of the electronic and photonic properties of these systems can be achieved by modulating the design of donor or acceptor units and connecting π-spacer units.^34–36^ Small organic π-conjugated donor–acceptor systems exhibit a low band gap, intense absorption, and strong intramolecular interactions.^37,38^ In several of these studies, triphenylamine, a classical nonplanar propeller shaped optoelectronic molecule, has been extensively used; extending their applications for developing field effect transistors, sensors, and solid state fluorescent and smart fluorescent materials.^39,40^

Tetracyanoethylene (TCNE) is a strong electron acceptor due to the presence of four cyano groups, and undergoes a [2 + 2] cycloaddition reaction with electron rich alkynes to form cyclobutene rings followed by a retroelectrocyclization reaction to give 1,1,4,4-tetracyanobutadiene (TCBD) derivatives.^41^ The donor–acceptor systems containing the TCBD acceptor are potential candidates for organic photovoltaics and non-linear optics due to strong intramolecular charge transfer (ICT) and lower HOMO–LUMO gaps.^42–48^ The photochemical behavior of a few donor-TCBD derived systems have been reported in the literature.^49–56^ Although with high quantum yields, due to close proximity between the donor and acceptor entities, ultrafast charge separation and recombination was observed in these systems. That is, no charge stabilization could be accomplished. In this regard, developing higher analogs of donor-TCBD bearing systems that would exhibit novel photochemical properties including charge stabilization has been scarce due to the associated synthetic challenges. A recent example involved *C*_3_-symmetric central truxene-derived phenothiazine–TCBD and its expanded molecular systems.^54^ Although elegant, the central truxene made exclusively of saturated carbons played no role in stabilizing the charge separated states.

**Chart 1:**
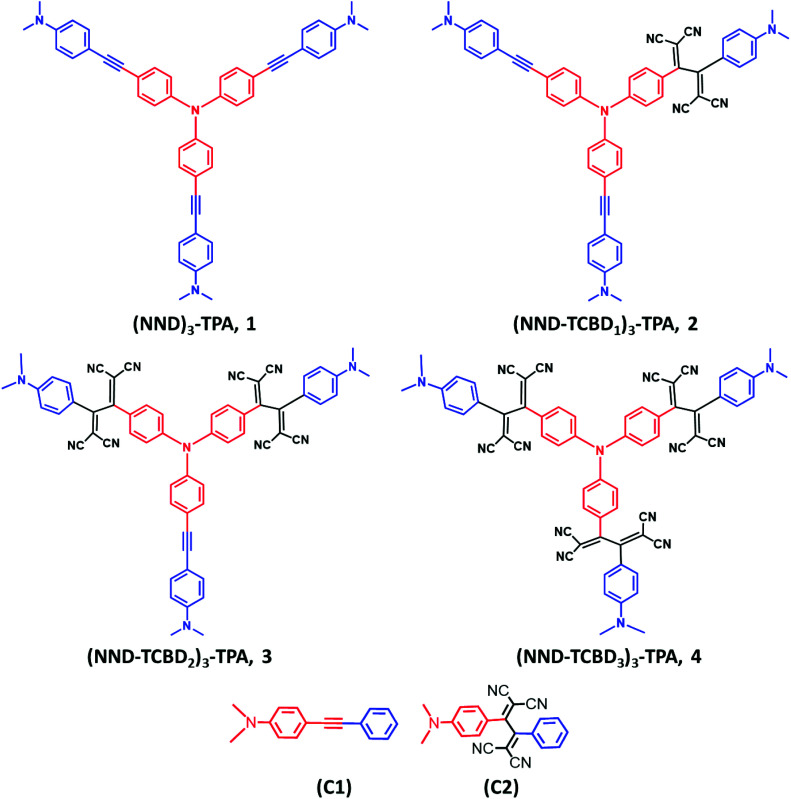
Structure and abbreviation of star-shaped, central triphenylamine derived, dimethylaminophenyl–tetracyanobutadiene conjugates, 1–4 and the control compounds, C1–C2 newly designed, synthesized to demonstrate charge stabilization *via* electron exchange in the present study.

In the present study, we hypothesize that by choosing a redox/photoactive central unit instead of truxene, we could modulate the properties that would lead to novel redox- and photo-chemical discoveries. With this in mind, we have newly designed and synthesized star-shaped symmetric compounds (NND)_3_-TPA, 1 and their TCBD functionalized symmetric and unsymmetric derivatives (NND–TCBD_1–3_)_3_–TPA, 2–4 (see Chart 1 for structures; NND = *N*,*N*-dimethylaminophenyl, TPA = triphenylamine and TCBD = 1,1,4,4-tetracyanobutadiene). These novel systems show strong intramolecular charge transfer (ICT) with lowered HOMO–LUMO gaps. Furthermore, upon electroreduction of NND–TCBD entities in compounds 3 and 4 containing two and three NND–TCBD entities, electron exchange between NND–TCBD was witnessed. Femtosecond transient absorption studies revealed the occurrence of ultrafast charge transfer processes in these systems. Importantly, charge stabilization in 3 and 4 to some extent was witnessed as a consequence of electron exchange. These unpresented new findings provide a new mechanism of stabilizing the charge separated states *via* electron exchange in multi-modular donor–acceptor conjugates.

## Results and discussion

**Scheme 1:**
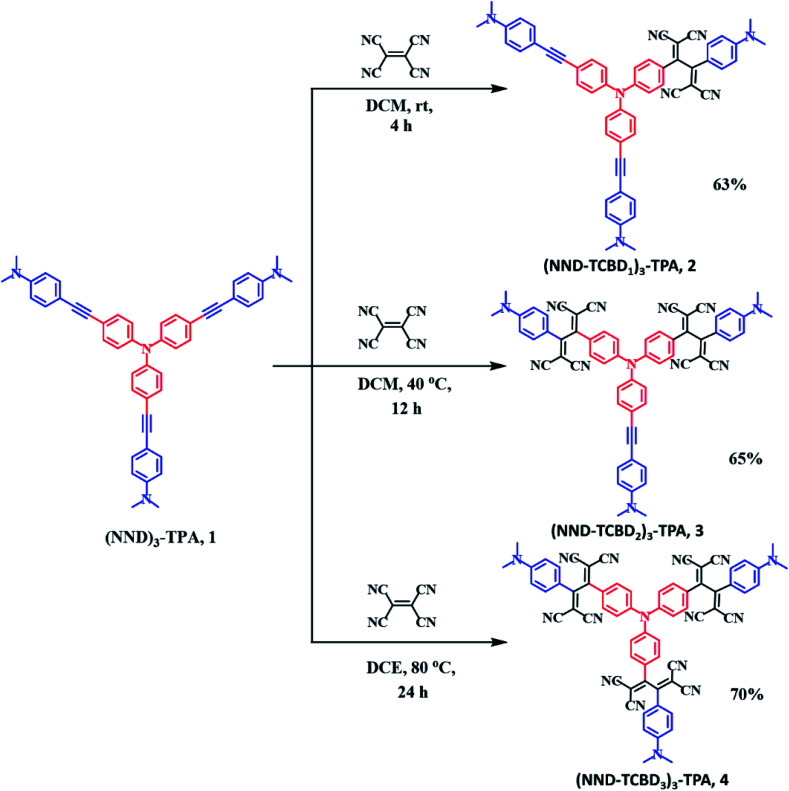
Synthetic scheme of compounds (NND)_3_–TPA 1, and (NND–TCBD_1–3_)_3_–TPA, 2–4.

Scheme 1 shows the developed synthetic scheme for compounds 1–4 and their controls. Briefly, the symmetric (NND)_3_–TPA, 1, was synthesized in 60% yield by the Pd-catalyzed Sonogashira cross coupling of tris-(4-iodo-phenyl)-amine and 4-ethynyl-*N*,*N*-dimethylaniline in degassed THF : TEA (1 : 1) under an argon atmosphere, in the presence of Pd(PPh_3_)_4_ and CuI. Next, (NND–TCBD_1–3_)_3_–TPA, 2–4, were synthesized *via* a [2 + 2] cycloaddition–retroelectrocyclization reaction with the strong electron acceptor TCNE. The reaction of 1 with one equivalent of TCNE in DCM at room temperature for 4 h resulted in an exclusive mono-TCBD bearing (NND–TCBD_1_)_3_–TPA, 2, in 63% yield. Similarly, the reaction of 1 with two equivalents of TCNE in DCM solvent at 40 °C for 12 h resulted in (NND–TCBD_2_)_3_–TPA, 3, in 65% yield, whereas increasing the reaction temperature to 80 °C in DCE solvent for 24 h using four equivalents of TCNE with 1 resulted in symmetrical (NND–TCBD_3_)_3_–TPA, 4, in 70% yield. The control compound C1 was synthesized by the Pd-catalyzed Sonogashira cross-coupling reaction of 4-ethynyl-*N*,*N*-dimethylaniline and iodobenzene in 60% yield. The acetylene linked control compound C1 was further subjected to a [2 + 2] cycloaddition-*retro*-electrocyclization reaction with one equivalent of TCNE at room temperature for 8 h, which resulted in TCBD substituted control compound C2 in 82% yield. The newly synthesized compounds were purified by silica gel (100–200 mesh) column chromatography using hexane:DCM as solvent and fully characterized by ^1^H, ^13^C NMR and high-resolution mass spectroscopy (HRMS) techniques (see the ESI for spectral details, Fig. S1–S18†).

**Fig. 1:**
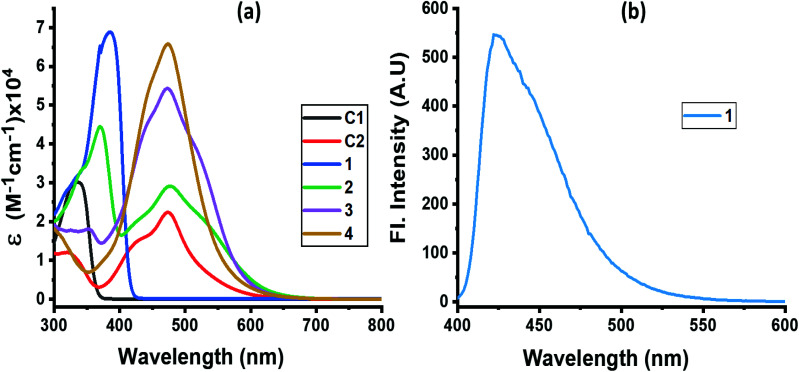
(a) Absorption and (b) fluorescence spectra of indicated compounds in DCB. Compound 1 was excited at 386 nm. No measurable emission was observed for compounds 2–4 upon exciting the samples at either the locally excited or charge transfer absorption peak positions.

The absorption spectrum of the investigated compounds is shown in Fig. 1a. Control C1, having only a NND entity without either TPA or TCBD entities, revealed an absorption band at 342 nm. In the case of control C2, having an electron acceptor, TCBD next to the electron donor, NND entity, promoted charge transfer interactions between them. Consequently, two peaks, the first one at 315 and a second broad peak corresponding to charge transfer absorption at 478 nm, was observed. Compound 1, having a central TPA and three terminal NND entities, revealed a single absorption peak at 386 nm. As predicted, no charge transfer type peak was present. However, in the case of compounds 2–4, having one, two and three TCBD entities between the NND and TPA entities, the expected charge transfer peak in the 478–484 nm region was possible to witness. In addition, a UV peak at 372 nm for 2, 350 nm for 3 and, and <300 nm for 4, respectively, was also observed. The intensity of the charge transfer band increased with increasing the number of TCBD entities. Due to spectral similarities between C2 and compounds 2–4, and enhanced absorption of the charge transfer band with an increase in TCBD, it was possible to conclude that the origin of the charge transfer band is primarily due to interaction between NND and TCBD entities with less contributions from TPA interaction with TCBD. Optical data are summarized in Table 1.

**Table 1:** Absorption and fluorescence, redox potentials (V *vs.* Ag/AgCl), and free-energy changes for charge recombination (CR), charge separation (CS) and charge transfer (CT) for the investigated central triphenylamine derived, dimethylamine–tetracyanobutadiene conjugates in DCB

System	*λ*_max_, nm	*E* (10^5^ M^−1^ cm^−1^)	*λ*_em_, nm	Red-2 TCBD	Red-1 TCBD	Ox-1 TPA	Ox-2 NND	Ox-3 NND	−Δ*G*_CR_	−Δ*G*_CS_	−Δ*G*_CT_
C1	337	2.99		—	—	—	0.96	—	—	—	—
C2	327, 473	1.16, 2.23		−0.73	−0.39	—	1.43	—	—	—	—
1	384	6.89	420	—	—	0.80	1.15	—	—	—	—
2	372, 477	4.40, 2.91	419	−0.69	−0.43	0.88	1.30	1.45	1.11	1.85	2.56
3	354, 472	1.80, 5.43	418	−0.73	−0.33^a^, −0.48	0.96	1.20	1.50	1.01	1.95	2.59
4	316, 476	1.63, 6.56	417	−0.70	−0.30^a^, −0.40	1.35	1.63	—	1.35	1.61	2.59

a Split peak.

Among the investigated compounds, only compound 1 revealed fluorescence emission as shown in Fig. 1b. A broad peak with maxima at 420 nm and a spectrum spanning the 400–575 nm range were observed (estimated quantum yield = 0.43). The single photon counting technique revealed a monoexponential decay for 1 with a lifetime of 1.16 ns. For compounds 2–4, having 1–3 strong electron acceptor TCBD entities, no measurable emission, at either the locally excited or charge transfer band positions, was observed; perhaps such emissions were too weak to detect. In any case, the strong quenching observed in the case of compounds 2–4 suggests the occurrence of excited state events such as energy or electron transfer in the highly interacting push–pull conjugates.

**Fig. 2:**
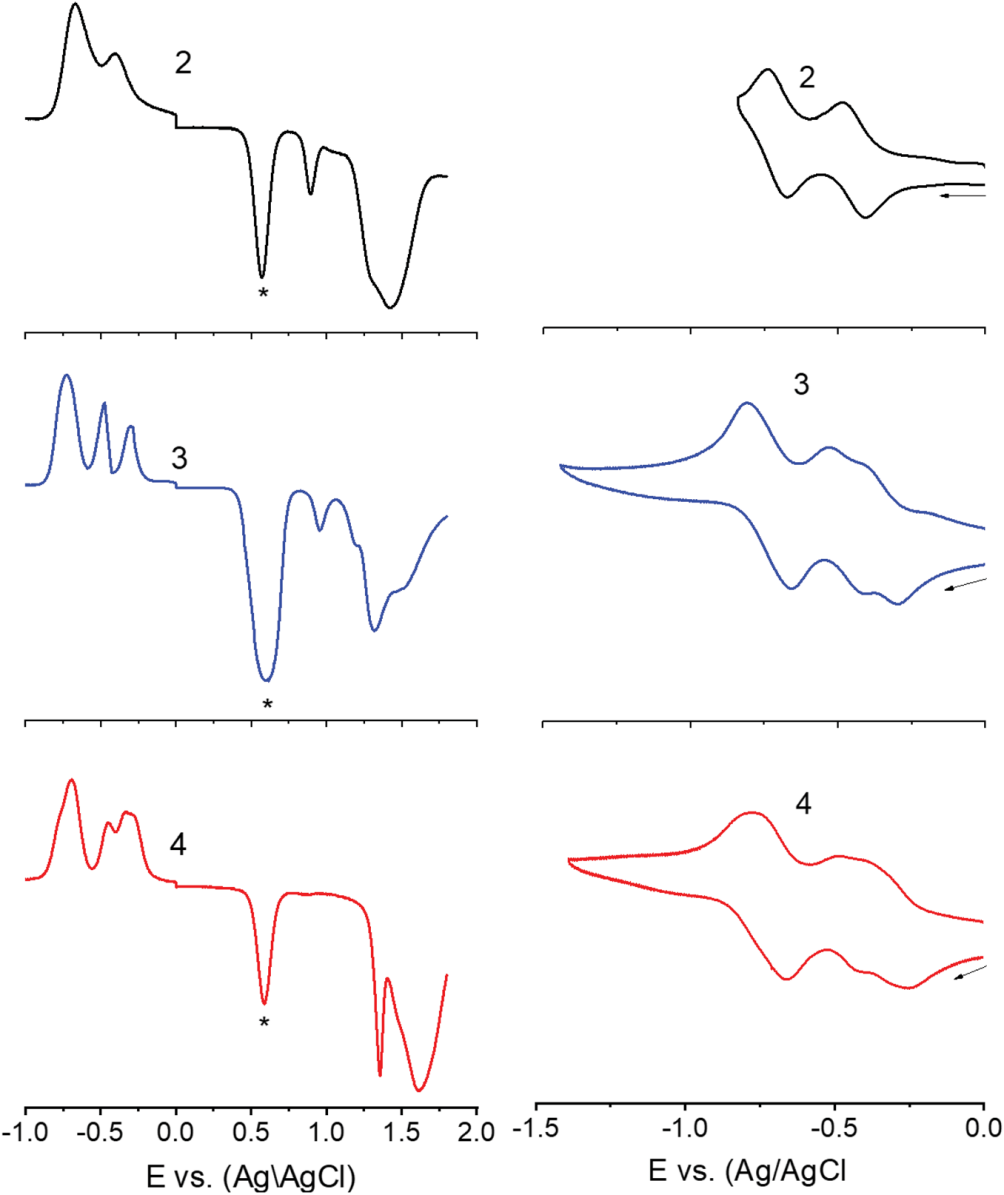
DPVs (left panel) and CVs (right panel) of indicated compounds in DCB containing 0.1 M (TBA)ClO_4_. For DPV: scan rate = 5 mV s^−1^, pulse width = 0.25 s, and pulse height = 0.025 V. For CV: scan rate = 100 mV s^−1^. The ‘*’ in the left panel represents the oxidation peak of ferrocene used as the internal standard. Note: the first reduction corresponding to TCBD in 3 and 4 is a split wave (see the text for details).

Next, in order to seek possible intramolecular interactions between the NND–TCBD entities *via* central TPA in compounds 3 and 4, electrochemical studies using differential pulse (DPV) and cyclic voltammetry (CV) were performed in DCB containing 0.1 M (TBA)ClO_4_. The site of electron transfer was arrived from control compounds C1 and C2 as summarized in Table 1 and representative voltammograms are shown in Fig. 2. Complete CVs are shown in Fig. S19 in the ESI.†

Key observations from electrochemical studies involved: (i) the first oxidation of C1 located at 0.96 V *vs.* Ag/AgCl was anodically shifted to 1.43 V in C2 due the presence of electron deficient TCBD. The TCBD reductions, all one-electron reversible, were located at −0.39 and −0.73 V. (ii) Compound 1 revealed two oxidations, the first one at 0.80 V and the second one at 1.15 V. From the peak currents and by comparison with the oxidation potential of C1, the first oxidation to TPA and the second one to NND entities were possible. (iii) In the case of compound 2, having a single NND–TCBD entity, the TPA oxidation was shifted to 0.88 V, while the NND oxidations were split and appeared at 1.30 and 1.45 V owing to the presence of two types of NNDs (one linked to TCBD and the other without TCBD). The TCBD reductions were located at −0.43 and −0.69 V. (iv) The introduction of a second TCBD entity into 3 and a third one into 4 revealed additional interesting features. As predicted, oxidation peaks revealed further anodic shift especially for TPA oxidation. Interestingly, the first reduction of TCBD in both compounds 3 and 4 was found to be split peaks. The split reduction peaks for 3 were located at −0.33 and −0.48 V, that is, a 140 mV potential difference while for 4, the split peaks were located at −0.30 and −0.40 V, that is, about a 100 mV potential difference. The second reduction of TCBD in both 3 and 4 was one-electron reduction without noticeable splitting. The splitting of the first reduction shows electron exchange between the NND–TCBD entities in 3 and 4*via* the central TPA entity.

**Fig. 3:**
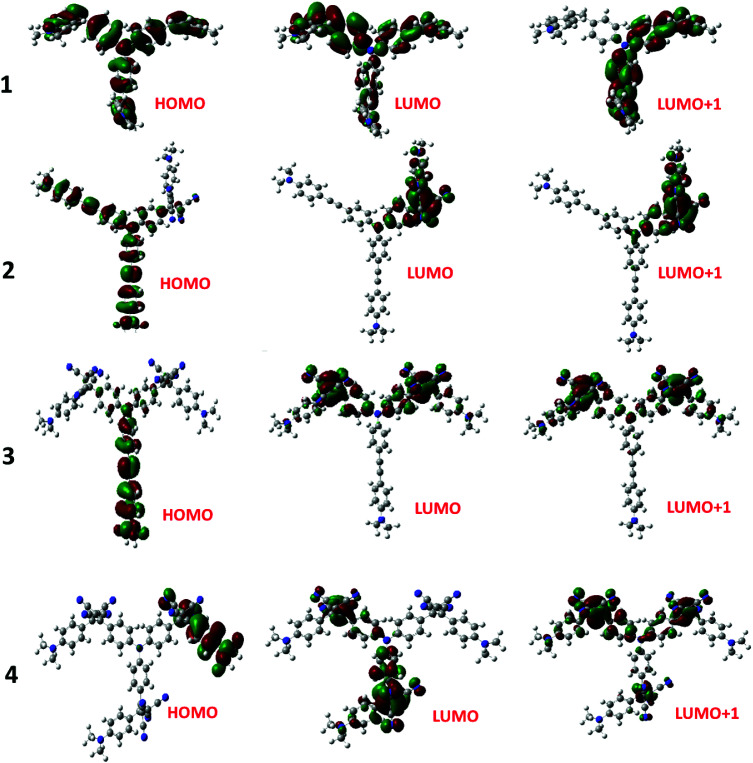
Frontier HOMO, LUMO and LUMO+1 of the investigated compounds from the B3LYP/6-31G** optimized structures (see the ESI for the coordinates of computed structures†).

The electron exchange between the NND–TCBD entities upon the first electroreduction of compounds 3 and 4 motivated us to perform computational studies to probe their electronic structures. Compounds 1–4 were fully optimized on a Born–Oppenheimer potential energy surface at the B3LYP/6-31G* level.^57^ The generated frontier orbitals on the optimized structures are shown in Fig. 3. The *C*_3_ type symmetry originating from the central TPA entity was obvious in all these compounds. In the case of 1, the HOMO was distributed evenly on the entire molecule while the LUMO coefficient was slightly more on one of the arms. In the case of 2, the HOMO was localized on NND–TPA arms, while the LUMOs were on the TCBD entity with some contributions extending into the NND and TPA entities. The energy difference between the two LUMOs was 0.0211 hartrees. In the case of compound 3, the HOMO occupied only the NND–TPA arm while the LUMOs occupied the two NND–TCBD entities with almost even distribution. The energy difference between the two LUMOs was 0.00527 hartrees. This situation was also true for compound 4, where the LUMO was distributed over two NND–TCBD entities while the LUMO+1 had contribution on all three NND–TCBD entities. The energy difference between the LUMOs was 0.0047 hartrees. The splitting of the first reduction peak due to electron exchange in the case of compounds 3 and 4 can now be attributed to energetically closely spaced LUMOs.

**Fig. 4:**
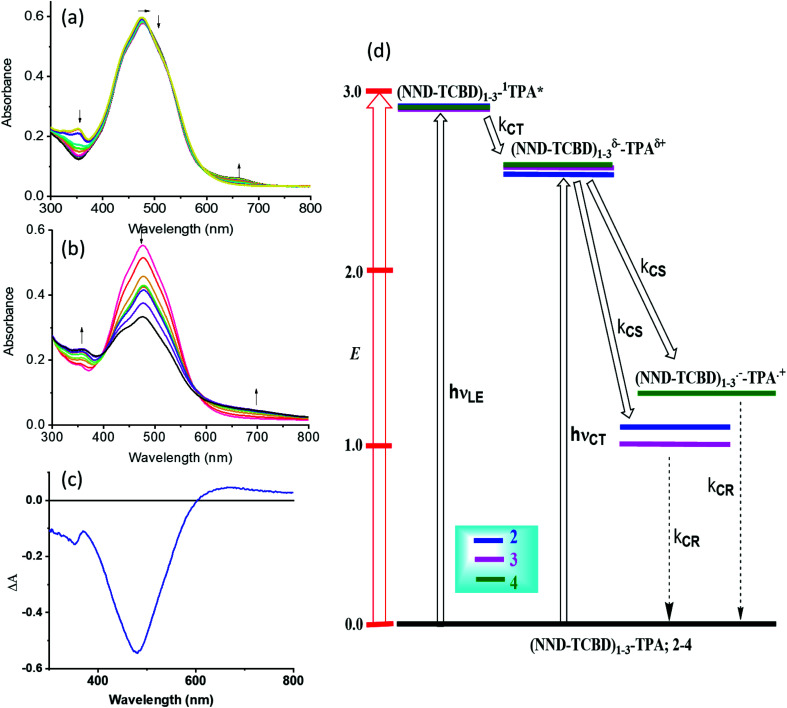
Spectral changes observed during the (a) first oxidation and (b) first reduction of 3 in DCB containing 0.2 M (TBA)ClO_4_. (c) Spectrum deduced for the charge separation state using spectroelectrochemical data (see the text for details, and Fig. S20 in the ESI† for complete results). (d) Energy level diagram showing possible charge transfer and charge separation events upon photoexcitation of the compounds 2–4. NND without linked TCBD in 2–3 is not shown in the abbreviated formula for simplicity.

One of the approaches to visualize the spectrum of charge separation products is by performing spectroelectrochemical studies followed by spectral interpretation. Here, by applying appropriate potentials corresponding to oxidation or reduction, the spectrum of the radical cation and radical anion can be generated. Subsequently, the average of the radical cation and radical anion spectrum will be digitally generated and subtracted from the spectrum of the neutral compound. This represents the differential absorption spectrum of the charge separation product. Positive peaks represent transitions associated with the electron transfer product, while negative peaks represent the depletion of the absorption of the neutral compound.^54^ We have used this approach in the present study as shown in Fig. 4a–c. Spectral changes associated with the first oxidation of 3 are shown in Fig. 4a. A new peak during the process of oxidation was observed at 662 nm. The spectral changes were minimal in the visible region as the main 478 nm peak was due to NND–TDCB charge transfer transition, whereas the oxidation was on the TPA entity (*vide supra*), ascertaining the earlier discussed site of electron transfer. In contract, the spectral changes during the first reduction (Fig. 4b) revealed a drastic decrease in the intensity of the charge transfer band with broad positive spectral features in the 600–800 nm range. It may be mentioned here that during both oxidation and reduction, no new peaks beyond 800 nm were observed. The spectrum generated for the charge transfer product using the above described procedure is shown in Fig. 4c. Such a spectrum revealed a positive peak at 670 nm and a depleted peak at 478 nm. Witnessing such a spectrum in transient absorption spectral studies would provide direct proof of charge transfer in these donor–acceptor conjugates. Similar spectra were derived for compounds 2 and 4 (see Fig. S20 in the ESI†).

An energy diagram was established to visualize the energetics of charge transfer and charge separation states in these conjugates, as shown in Fig. 4d. The energy of different states was established from free-energy calculations,^58,59^ as listed in Table 1. From such a diagram, it was clear that the excitation of the conjugates, 2–4, at either the locally excited (near-UV) or charge transfer (visible) peak positions would produce the respective excited states. The excited state species, (NND–TCBD)_1–3_–^1^TPA*, formed from the locally excited state would readily produce the initial charge transfer state, (NND–TCBD)_1–3_^*δ*−^–TPA^*δ*+^, involving one of the NND–TCBD entities (free NND is not abbreviated for simplicity). Such a state can also be produced by direct excitation of the visible charge transfer band. The charge transfer state thus generated could further undergo electron transfer to generate the (NND–TCBD_1–3_)˙^−^–TPA˙^+^ charge separated species. Although the initial charge separation state would involve only one of the NND–TCBD entities, due to electron exchange, the anion radical could spread over other NND–TCBD entities, as suggested by the earlier discussed frontier LUMOs. Finally, the charge separated species could relax back to the ground state.

In order to probe the anticipated photochemical events and to seek the effect of multiple NND–TCBD entities in prolonging the lifetime of charge separated states *via* the earlier discussed electron exchange mechanism, femtosecond transient absorption studies (fs-TA) were performed. Three solvents of varying polarity were used as the solvent polarity would influence the lifetime of charge separated states, and samples were excited at both locally excited (350 nm) and charge transfer (500 nm) peak positions.

**Fig. 5:**
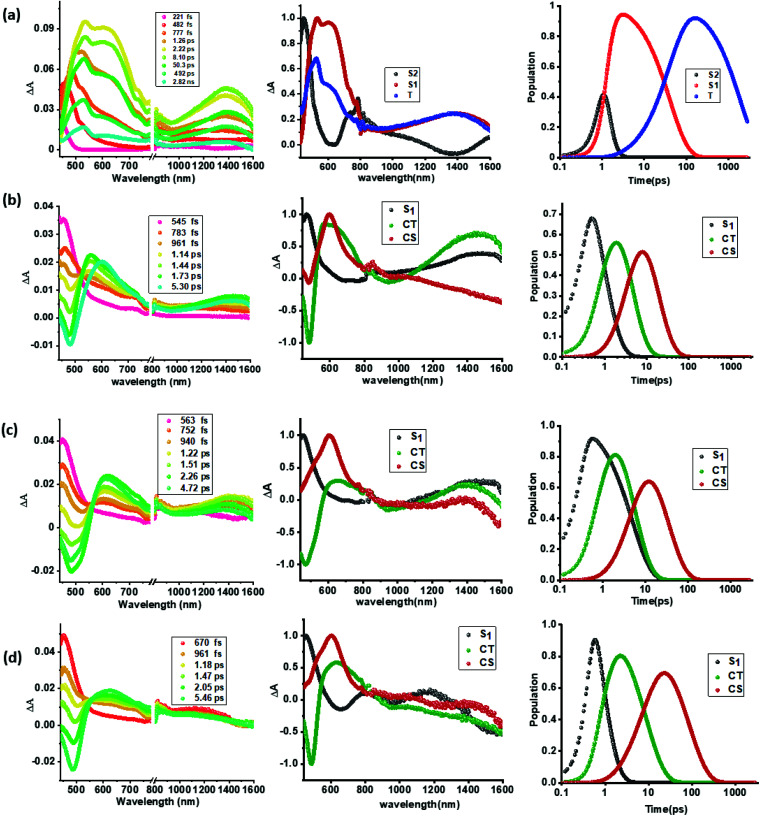
fs-TA spectra at the indicated delay times, (a–d, panel i), species associated spectra (a–d, panel ii), and population kinetics (a–d, panel iii) of compounds 1–4 (a through d) in benzonitrile. The samples were excited at 350 nm corresponding to the locally excited state.

As shown in Fig. 5a(i), a singlet excited state of compound 1 (^1^TPA*) in benzonitrile was formed instantaneously upon 350 nm laser excitation featuring excited state absorption (ESA) maxima at 533, 601 and 1382 nm (see the spectrum at 2.22 ps) as well as ground state bleaching in the 400–450 nm range. To gather insight into the deactivation, global target analysis^60,61^ was performed. A kinetic model including three species was satisfactory. The species associated spectra (SAS) and population kinetics of the three species are shown in Fig. 5a(ii and iii), respectively. The first species with a lifetime of ∼200 fs was within the temporal resolution of our instrument that decayed to develop the second component with singlet excited state features with a lifetime of 49 ps. The third component which could be attributed to the decaying singlet to triplet state had a time constant of about 2.30 ns. Due to the lack of any TCBD entities in 1, no electron transfer could be detected.

In contrast to the spectral features of compound 1, the donor–acceptor conjugates 2–4 revealed the anticipated ultrafast charge transfer and charge separation processes. In the case of 2, where only one NND–TCBD entity is present, the occurrence of relatively simple photoinduced charge transfer could be envisioned. However, in the case of 3 and 4 featuring two and three entities of NND–TCBD attached to TPA, a symmetry breaking charge transfer could be envisioned due to the presence of multiple numbers of equally positioned acceptor entities. The presence of a higher number of acceptors could improve the charge transfer by the respective statistical factor or even more by quantum coherence effects.^62^ The first panel in Fig. 5b–d shows transient spectra at the indicated delay times for compounds 2–4. The ESA peak of the singlet excited state located in the 450–500 nm range revealed a rapid decay with new peaks in the 610–620 nm range and near-IR range. These spectra were subjected to target analysis that required three components for a satisfactory fit. These SAS are shown in Fig. 5b–d, middle panel. In all these spectra, the first spectrum with characteristic features of the singlet excited state had a time constant of less than 1 ps, and as predicted, the magnitude of these time constants further decreased with increase in the number of NND–TCBD entities (statistical factor of quenching). The second SAS with time constants of 3.48–5.64 ps have been attributed to the charge transfer state. Here, the depleted peak intensity in the near-IR region has been tentatively assigned to stimulated emission of the CT state. With the decay of the second component, the third component was evolved which has been attributed to the charge separated species as this spectrum resembled largely that derived for the charge separation product from the earlier discussed spectroelectrochemical studies (see Fig. 4c). It may be mentioned here that the SAS of the third component are distinctly different from the triplet state SAS shown in Fig. 5a, panel (ii). The time constants for the charge separated state were found to be 14.9, 32.68 and 75.1 ps, respectively, for compounds 2, 3, and 4. These results reveal the persistence of the charge separated state in compounds 3 and 4 compared to that in 2.

**Fig. 6:**
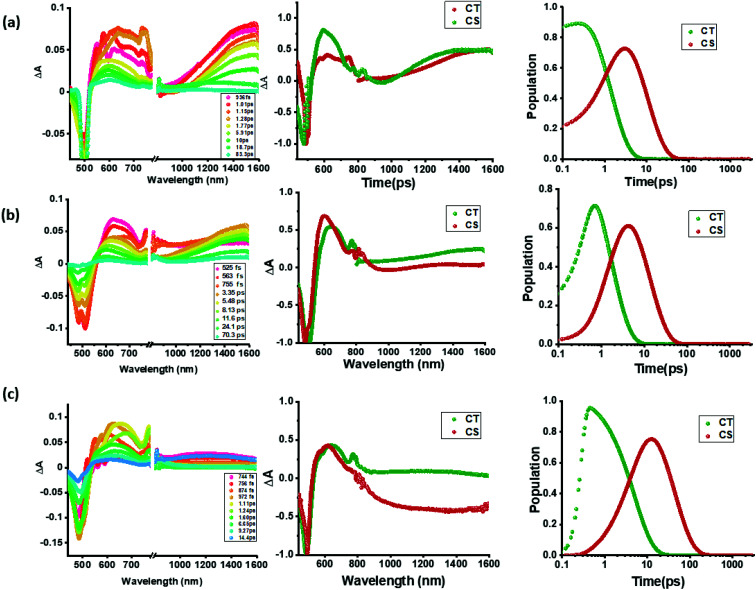
fs-TA spectra at the indicated delay times of compounds 2–4 in benzonitrile. The samples were excited at 500 nm corresponding to the charge transfer band. Right hand panel shows the population kinetics. The dip at 500 nm is due to the excitation laser.

Intrigued by these findings, next, we changed the solvent to less polar DCB and nonpolar toluene. In both of these solvents the spectral trends were almost the same (see Fig. S21 and S22 in the ESI†). Furthermore, target analysis was performed to evaluate the kinetic factors as listed in Table 2. Such data confirmed the persistence of charge separated states in both solvents. Changing the excitation wavelength to 500 nm corresponding to the charge transfer also revealed excited state charge separation (Fig. 6, S23 and S24†). In this case, the data could be satisfactorily fitted to two components, one to the excited state charge transfer with time constants of a few ps and the second one for the charge separated state. It may be mentioned here that irrespective of the excitation wavelengths (LE or CT), the SAS generated for charge transfer and charge separation states revealed close resemblance.

**Table 2:** Time constants evaluated from GloTarAn target analysis of fs-TA spectral data in solvents of varying polarity and at different excitation wavelengths for the investigated central triphenylamine derived, dimethylamine–tetracyanobutadiene conjugates

Compound	Solvent	*λ*_ex_, nm	S_1_, ps	CT, ps	CS, ps
1	Toluene	350	842	—	—
2			2.36	4.17	17.02
3			1.56	10.01	39.32
4			0.54	25.37	93.20
2		500	—	3.59	14.92
3			—	7.29	24.10
4			—	16.8	56.30
1	DCB	350	111	—	—
2			1.99	3.87	16.40
3			1.14	8.12	37.59
4			0.35	13.6	87.70
2		500	—	2.49	13.93
3			—	5.78	20.45
4			—	8.76	60.41
1	PhCN	350	49	—	—
2			0.89	3.48	14.9
3			0.80	4.99	32.68
4			0.67	5.64	75.10
2		500	—	1.38	10.23
3			—	3.69	24.65
4			—	4.98	43.29

As pointed out earlier, in synthetic multimodular donor–acceptor systems, charge stabilization is often achieved by following the mechanism of natural photosynthesis where electron migration occurs across the system distantly separating the positive and negative ions thus minimizing their electrostatic attraction.^20–22^ Electron/hole delocalization in multiple donor or accepting bearing systems, and utilization of high-energy triplet sensitizers to promote electron transfer from the long-lived triplet excited states are also some of the known mechanisms to extend the lifetime of the charge separated species. The present multi-modular systems, 3 and 4, differ in their design, wherein the same acceptor unit, NND–TCBD, is covalently linked to the central TPA. Electron exchange has been witnessed upon the first reduction of these compounds unlike that in 2 bearing a single NND–TCBD entity. It appears that such electron exchange is responsible for extending the lifetime of the charge separated states in these novel donor–acceptor conjugates.

## Conclusions

In summary, we have developed exceptional molecular donor–acceptor systems consisting of *C*_3_ symmetric central triphenylamine derived, dimethylamine–tetracyanobutadiene conjugates. In these systems, NND–TCBD promoted charge transfer extending the absorption covering the visible region. Electrochemical studies revealed electron exchange in compounds 3 and 4 carrying multiple numbers of NND–TCBD entities. Frontier LUMO energy levels and orbital coefficients helped us in rationalizing such electron exchanges. The spectrum of the charge transfer state was possible to deduce from the manipulation of spectroelectrochemical data. Finally, we have been able to demonstrate the effect of electron exchange in prolonging the lifetime of charge separated states in compounds 3 and 4 by fs-TA spectral studies in solvents of varying polarity. To our knowledge, this is the first report where such a charge stabilizing mechanism involving electron exchange has been proposed and demonstrated experimentally. The present findings are very important to further our understanding on the fundamentals of electron transfer in multi-modular systems, strengthen our knowledge on the early events of natural photosynthesis, and seek novel applications in optoelectronics. In this context, it may be pointed out here that in bacterial photosynthesis, the primary electron donor is an electronically interacting bacteriochlorophyll dimer, [BChl]_2_.^63^ The initial electron transfer species, [BChl]_2_˙^+^ could slow down the charge recombination *via* a similar electron exchange mechanism and could be a reason for the natural choice of a bacteriochlorophyll dimer instead of a monomer.

## Conflicts of interest

There are no conflicts to declare.

## Supplementary Material

SC-012-D0SC04648E-s001
