# Supplementary material for: Charge stabilization via electron exchange: excited charge separation in symmetric, central triphenylamine derived, dimethylaminophenyl–tetracyanobutadiene donor–acceptor conjugates
Source: Chem Sci. 2020 Nov 13;12(3):1109–20. doi: 10.1039/d0sc04648e (PMC8179009; doi:10.1039/d0sc04648e)
Supplement: SC-012-D0SC04648E-s001 [file SC-012-D0SC04648E-s001.pdf]

Electronic Supplementary Material (ESI) for Chemical Science.
This journal is © The Royal Society of Chemistry 2020

Electronic Supplemental Information

Charge Stabilization via Electron Exchange: Excited Charge Separation in Symmetric, Central Triphenylamine Derived, Dimethylaminophenyl-Tetracyanobutadiene Donor-Acceptor Conjugates

Indresh S. Yadav,[a,‡] Ajyal Z. Alsaleh,[b,‡] Rajneesh Misra[a,*] and Francis D'Souza[b,*]

[a]Department of Chemistry, Indian Institute of Technology, Indore 453552, India. E-mail: rajneeshmisra@iiti.ac.in

[b]Department of Chemistry, University of North Texas, 1155 Union Circle, #305070, Denton, TX 76203-5017, USA, E-mail: Francis.DSouza@UNT.edu

Experimental Section

General methods

All the chemicals were used as received unless otherwise indicated. All oxygen or moisture sensitive reactions were performed under inert atmosphere. All the chemicals were purchased from commercial sources and used without further purification. ^{1}H NMR (400 MHz), and ^{13}C NMR (100MHz) spectra were recorded on the Bruker Avance (III) 400 MHz, using $CDCl_3$ as solvent and the chemical shifts were reported in parts per million (ppm) with TMS (0 ppm) and $CDCl_3$ (77.00) as standards. Tetramethylsilane (TMS) was used as reference for recording ^{1}H (of residual proton; δ = 7.26 ppm), and ^{13}C (δ = 77.0 ppm) spectra in $CDCl_3$. HRMS was recorded on Brucker-Daltonics, micrO TOF-Q II mass spectrometer.

The UV-visible spectral measurements were carried out with a Shimadzu Model 2550 double monochromator UV-visible spectrophotometer. The fluorescence emission was monitored by using a Horiba Yvon Nanolog coupled with time-correlated single photon counting with nanoLED excitation sources. A right angle detection method was used. Differential pulse and cyclic voltammograms were recorded on an EG&G PARSTAT electrochemical analyzer using a three electrode system. A platinum button electrode was used as the working electrode. A platinum wire served as the counter electrode and an Ag/AgCl electrode was used as the reference electrode. Ferrocene/ferrocenium redox couple was used as an internal standard. All the solutions were purged prior to electrochemical and spectral measurements using argon gas.

Femtosecond transient absorption spectroscopy experiments were performed using an ultrafast femtosecond laser source (Libra) by Coherent incorporating a diode-pumped, modelocked Ti:sapphire laser (Vitesse) and a diode-pumped intracavity doubled Nd:YLF laser (Evolution) to generate a compressed laser output of 1.45 W. For optical detection, a Helios transient absorption spectrometer coupled with a femtosecond harmonics generator, both provided by Ultrafast Systems LLC, was used. The sources for the pump and probe pulses were derived from the fundamental output of Libra (Compressed output 1.45 W, pulse width 100 fs) at a repetition rate of 1 kHz; 95% of the fundamental output of the laser was introduced into a TOPAS-Prime-OPA system with a 290−2600 nm tuning range from Altos Photonics Inc., (Bozeman, MT), while the rest of the output was used for generation of a white light continuum. Kinetic traces at appropriate

wavelengths were assembled from the time-resolved spectral data. Data analysis was performed using Surface Xplorer software supplied by Ultrafast Systems. All measurements were conducted in degassed solutions at 298 K. The estimated error in the reported rate constants is ±10%.

Synthetic route for Triphenylamine Derivative 1 ((NND)$_3$-TPA, 1):

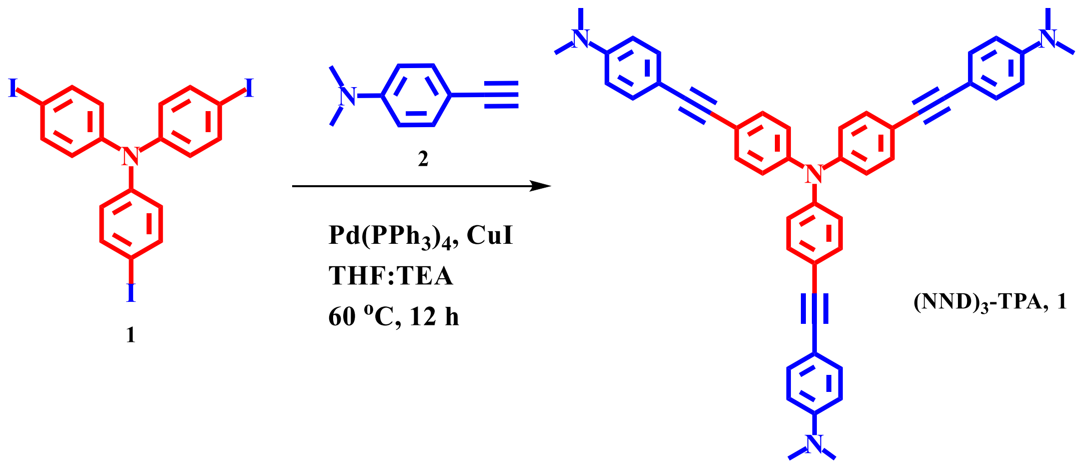

Synthetic procedure of Triphenylamine Derivative 1 ((NND)$_3$-TPA, 1) and Triphenylamine Derivative 2–4 ((NND-TCBD$_{1-3}$)$_3$-TPA, 2–4):

Triphenylamine Derivative 1 ((NND)$_3$-TPA, 1): Under argon atmosphere a solution of tris-(4-iodo-phenyl)-amine (**1**) (0.5 g, 0.80 mmol) and the corresponding 4-ethynyl-*N*,*N*-dimethylaniline (**2**) (0.466 g, 3.2 mmol) in dry THF (50 ml), added triethylamine (50 ml), Pd(PPh$_3$)$_4$ (0.046 g, 0.04 mmol), CuI (0.007 g, 0.04 mmol), stirred for 12 h at 60 ^oC, after completion of the reaction, the reaction mixture was concentrated under reduced pressure, the crude compound was purified by column chromatography on silica, using Hexane/ DCM (60:40, v/v), and afforded pure compound **(NND)$_3$-TPA, 1** around 60 % yield. Brown solid (0. 320 g, 60 %). **^{1}H NMR (CDCl$_3$, 400 MHz, ppm):** δ 7.40-7.38 (m, 12H), 7.04 (d, *J* = 8 Hz, 6H), 6.66 (d, *J* = 12 Hz, 6H), 2.99 (s, 18H). **^{13}C NMR (CDCl$_3$, 100 MHz, ppm):** 149.9, 146.1, 132.6, 132.3, 123.9, 118.6, 111.8, 110.2, 90.2, 87.2, 40.2; **HRMS (ESI, positive)** m/z calculated for $C_{48}H_{42}N_4$ 675.3482 [M + nH] $^+$, measured 675.3489 [M + nH] $^+$.

Triphenylamine Derivative 2 ((NND-TCBD$_1$)$_3$-TPA, 2): In a 50 mL round bottomed flask, tetracyanoethylene (TCNE, 18 mg, 0.1 mmol) was added to solution of **(NND)$_3$-TPA, 1** (100 mg, 0.1 mmol) in DCM (20 mL). The reaction mixture was stirred in room temperature for 4 h. After completion of reaction, the reaction mix. was dried under vacuum and purified by column

chromatography with hexane/DCM (40:60, v/v) as eluent to give compound **(NND-TCBD$_1$)$_3$-TPA, 2** as dark red solid (Yield: 74 mg, 63 %). **^{1}H NMR (400 MHz, CDCl$_3$):** δ 7.80 (d, *J* = 8 Hz, 2H), 7.68 (d, *J* = 8 Hz, 2H), 7.49 (d, *J* = 8 Hz, 4H), 7.41 (d, *J* = 8 Hz, 4H), 7.13 (d, *J* = 8 Hz, 4H), 7.02 (d, *J* = 8 Hz, 2H), 6.72 (d, *J* = 8 Hz, 2H), 6.67 (d, *J* = 8 Hz, 4H), 3.17 (s, 6H), 3.01 (s, 12H). **^{13}C NMR (CDCl$_3$, 100 MHz, ppm):** δ 150.2, 143.6, 132.7, 132.5, 131.8, 126.1, 122.0, 119.4, 111.8 109.6, 91.7, 86.6, 53.4, 40.1; **HRMS (ESI, positive)** m/z calculated for $C_{54}H_{42}N_8$ 803.3605 [M + nH] $^+$, measured 803.3606 [M + nH] $^+$.

Triphenylamine Derivative 3 ((NND-TCBD$_2$)$_3$-TPA, 3): In a 50 mL round bottomed flask, tetracyanoethylene (TCNE, 37 mg, 0.29 mmol) was added to solution of **(NND)$_3$-TPA, 1** (100 mg, 0.14 mmol) in DCM (20 mL). The reaction mixture was heated at 40 ^{o}C for 12 h. After completion of reaction, the reaction mix. was dried under vacuum and purified by column chromatography with hexane/DCM (20:80, v/v) as eluent to give **(NND-TCBD$_2$)$_3$-TPA, 3** as dark red solid (Yield: 89 mg, 65 %). **^{1}H NMR (400 MHz, CDCl$_3$):** δ 7.79 (d, *J* = 8 Hz, 3H), 7.70 (d, *J* = 8 Hz, 4H), 7.53 (d, *J* = 8 Hz, 1H), 7.47-7.39 (m, 3H), 7.36-7.33 (m, 1H), 7.18-7.12 (m, 7H), 6.74 (d, *J* = 8 Hz, 4H), 6.66 (d, *J* = 8 Hz, 1H), 3.17 (s, 12H), 3.00 (s, 6H). **^{13}C NMR (CDCl$_3$, 100 MHz, ppm):** δ 166.8, 163.4, 154.46, 150.9, 150.7, 150.3, 143.8, 142.7, 133.2, 132.8, 132.4, 131.5, 131.5, 130.6, 123.0, 127.7, 127.6, 126.4, 126.1, 112.2, 111.7, 109.3, 83.4, 74.2, 40.1; **HRMS (ESI, positive)** m/z calculated for $C_{60}H_{42}N_{12}$ 953.3548 [M + Na] $^+$, measured 953.3548 [M + Na] $^+$.

Triphenylamine Derivative 4 ((NND-TCBD$_3$)$_3$-TPA, 4): In a 50 mL round bottomed flask, tetracyanoethylene (TCNE, 75 mg, 0.59 mmol) was added to solution of **(NND)$_3$-TPA, 1** (100 mg, 0.14 mmol) in DCE (20 mL). The reaction mixture was heated at 80 ^{o}C for 24 h. After completion of reaction, the reaction mix. was dried under vacuum and purified by column chromatography with hexane/DCM (10:90, v/v) as eluent to give **(NND-TCBD$_3$)$_3$-TPA, 4** as dark red solid (Yield: 110 mg, 70 %). **^{1}H NMR (400 MHz, CDCl$_3$):** δ 7.82 (d, *J* = 8 Hz, 6H), 7.74 (d, *J* = 8 Hz, 6H), 7.23 (d, *J* = 8 Hz, 6H), 6.77 (d, *J* = 8 Hz, 6H), 3.20 (s, 18H). **^{13}C NMR (CDCl$_3$, 100 MHz, ppm):** δ 167.0, 162.7, 154.5, 149.6, 132.4, 131.6, 128.1, 124.9, 117.7, 114.2, 113.5, 112.4, 112.3, 111.4, 85.3, 73.6, 40.1; **HRMS (ESI, positive)** m/z calculated for $C_{66}H_{42}N_{16}$ 1081.3671 [M + Na] $^+$, measured 1081.3672 [M + Na] $^+$.

Synthetic procedure of control compound C1 and C2:

***N,N*-dimethyl-4-(phenylethynyl)aniline (C1):** Under argon atmosphere a solution of 4-ethynyl-*N,N*-dimethylaniline **2** (0.1 g, 0.68 mmol) and the corresponding iodobenzene (**3**) (0.14 g, 0.68 mmol) in dry THF (50 ml), added triethylamine (20 ml), $Pd(PPh_3)_4$ (0.039 g, 0.03 mmol), CuI (0.006 g, 0.03 mmol), stirred for 12 h at 60 ^oC, after completion of the reaction, the reaction mixture was concentrated under reduced pressure, the crude compound was purified by column chromatography on silica, using Hexane/ DCM (90:10, v/v), and afforded pure compound **C1** around 60 % yield. White Brown solid (0.102 g, 67 %). **^{1}H NMR ($CDCl_3$, 400 MHz, ppm):** δ 7.50 (d, *J* = 8 Hz, 2H), 7.41 (d, *J* = 8 Hz, 2H), 7.34-7.27 (m, 3H), 6.86 (d, *J* = 8 Hz, 2H), 2.99 (s, 6H). **^{13}C NMR ($CDCl_3$, 100 MHz, ppm):** 150.10, 132.70, 131.28, 128.22, 127.43, 124.14, 111.84, 110.07, 90.58, 87.32, 40.22; **HRMS (ESI, positive)** m/z calculated for $C_{16}H_{15}N$ 222.1277 [M + nH] $^+$, measured 222.1278 [M + nH] $^+$.

2-(4-(dimethylamino)phenyl)-3-phenylbuta-1,3-diene-1,1,4,4-tetracarbonitrile (C2): In a 50 mL round bottomed flask, tetracyanoethylene (TCNE, 57 mg, 0.45 mmol) was added to solution of compound **C1** (100 mg, 0.14 mmol) in DCM (20 mL). The reaction mixture was stirred for 24 h at room temperature. After completion of reaction, the reaction mix. was dried under vacuum and purified by column chromatography with hexane/DCM (30:70, v/v) as eluent to give compound **C2** as dark red solid (Yield: 130 mg, 82 %). **^{1}H NMR (400 MHz, $CDCl_3$):** *δ* 7.79 (d, *J* = 8 Hz, 2H), 7.73 (d, *J* = 8 Hz, 2H), 7.63 (t, *J* = 8 Hz, 1H), 7.54 (t, *J* = 8 Hz, 2H), 6.74 (d, *J* = 8 Hz, 2H), 3.17 (s, 6H). **^{13}C NMR ($CDCl_3$, 100 MHz, ppm):** *δ* 169.32, 163.24, 154.41, 134.25, 132.50, 131.95, 129.73, 129.48, 117.98, 114.24, 113.30, 112.25, 112.01, 111.25, 87.21, 74.40, 40.16; **HRMS (ESI, positive)** m/z calculated for $C_{22}H_{15}N_5$ 350.1400 [M + nH] $^+$, measured 350.1391 [M + nH] $^+$.

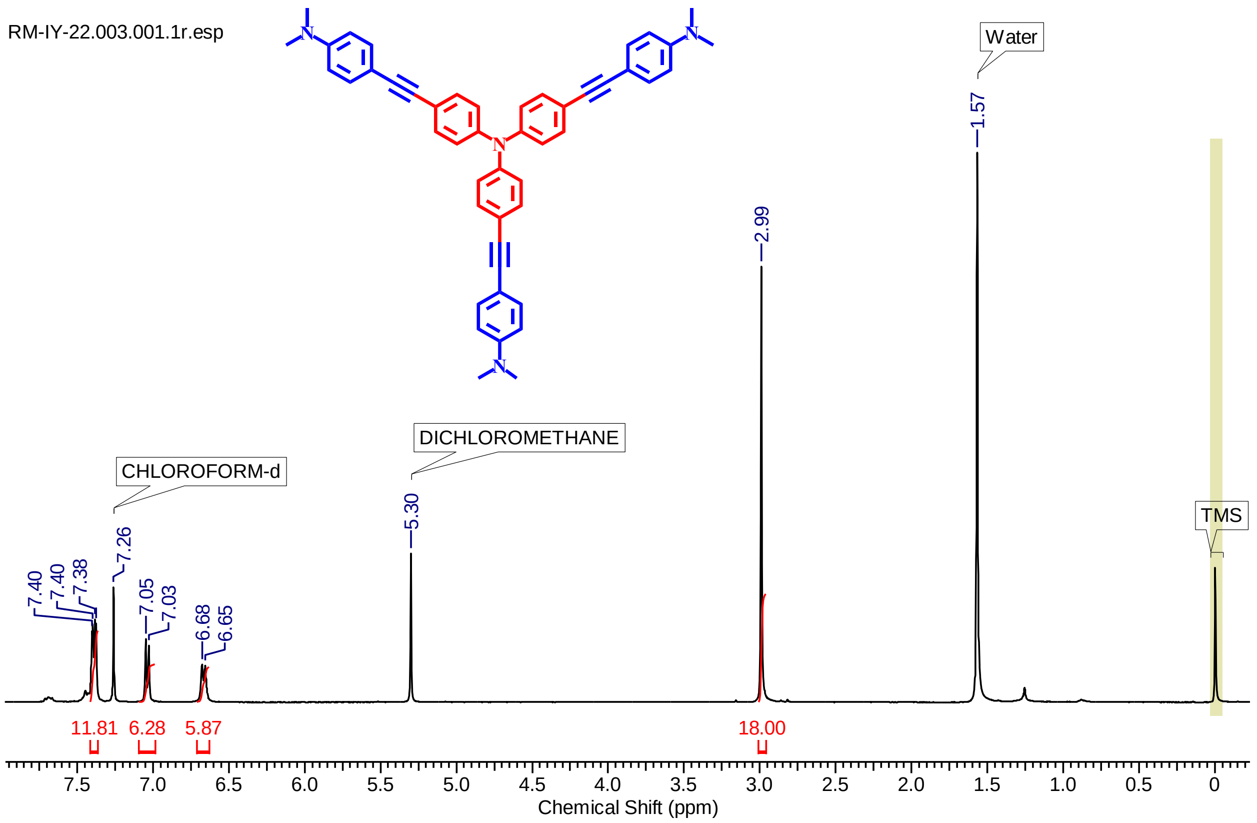

Figure S1. ^{1}H NMR Spectra of **(NND)$_3$-TPA, 1.**

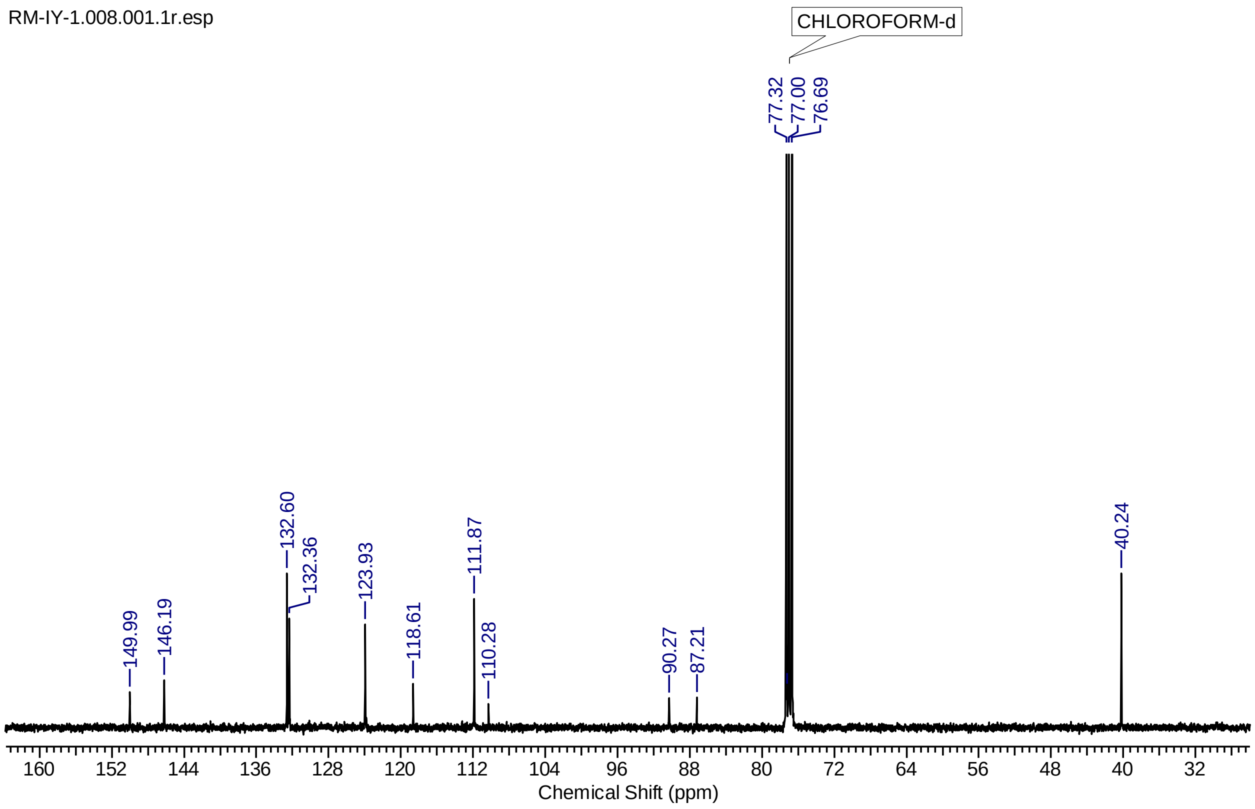

Figure S2. ^{13}C NMR Spectra of **(NND)$_3$-TPA, 1.**

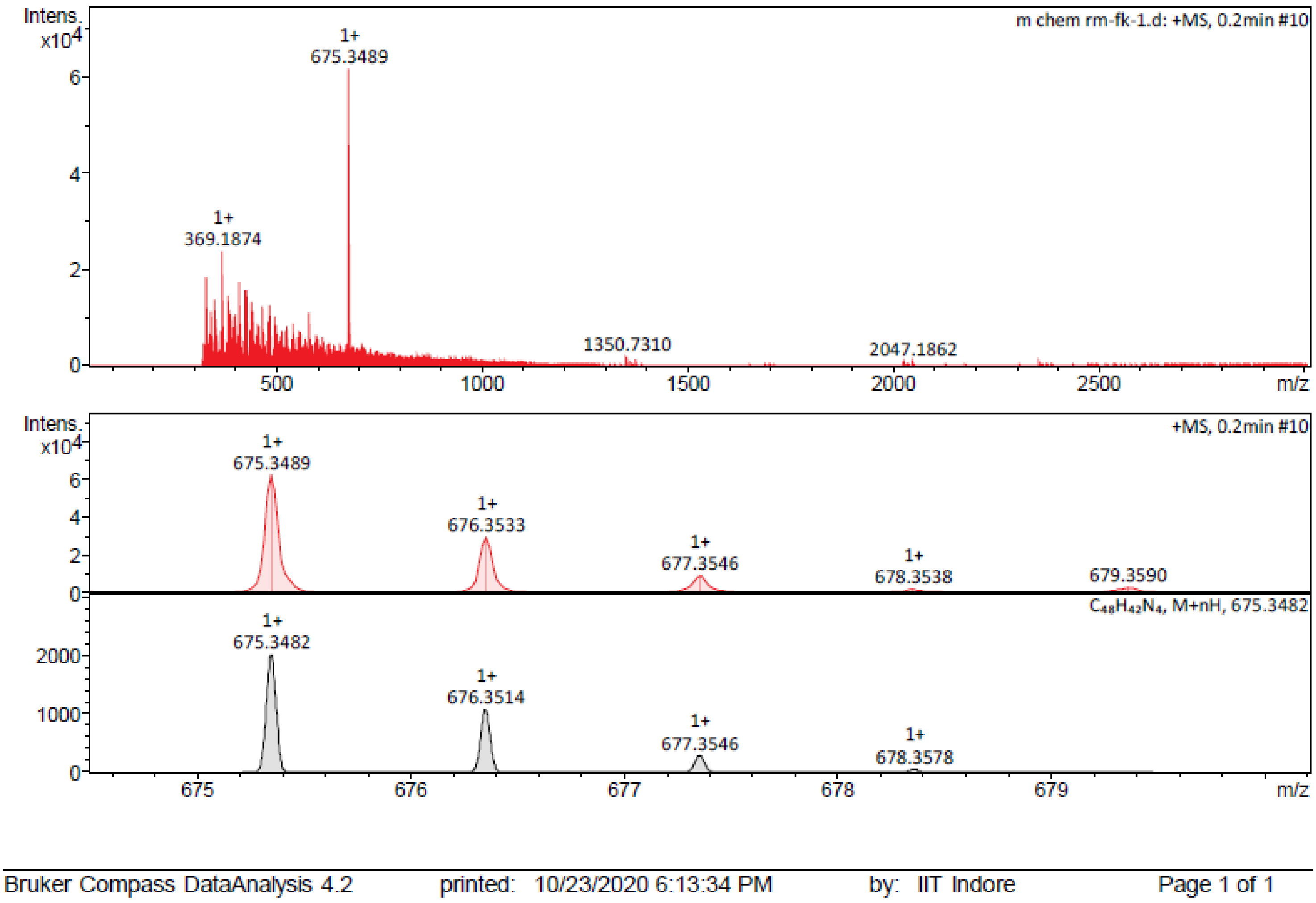

Figure S3. HRMS Spectra of **(NND)$_3$-TPA, 1.**

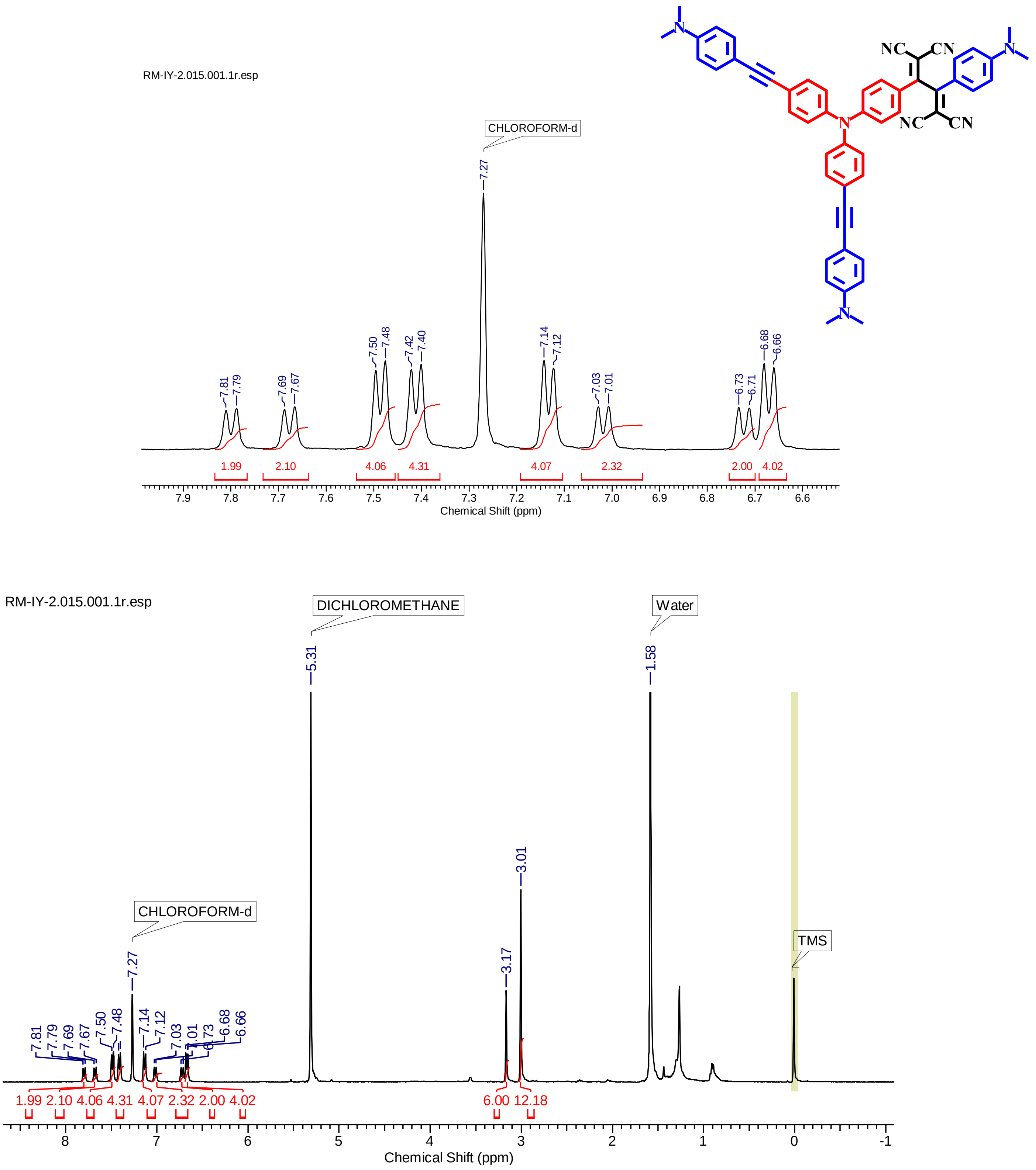

Figure S4. ^{1}H NMR Spectra of **(NND-TCBD$_1$)$_3$-TPA, 2.**

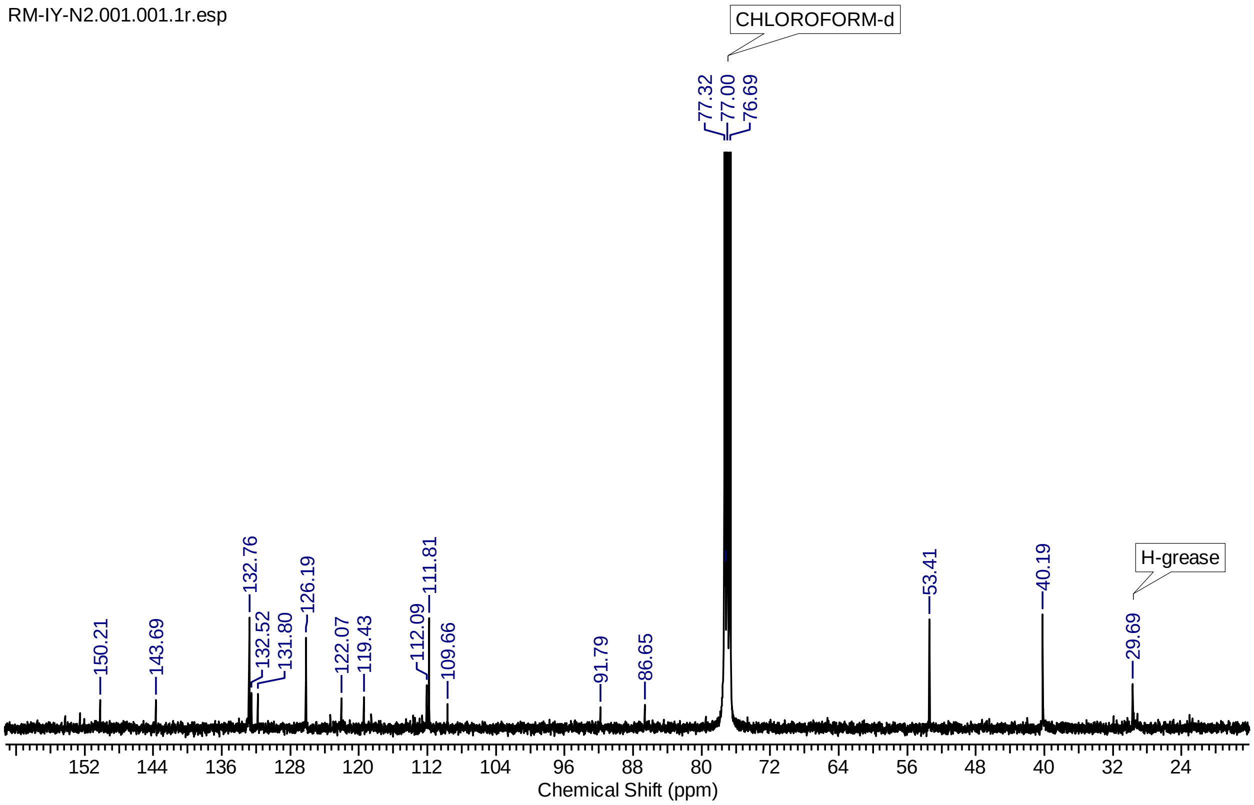

Figure S5. ^{13}C NMR Spectra of **(NND-TCBD$_1$)$_3$-TPA, 2**.

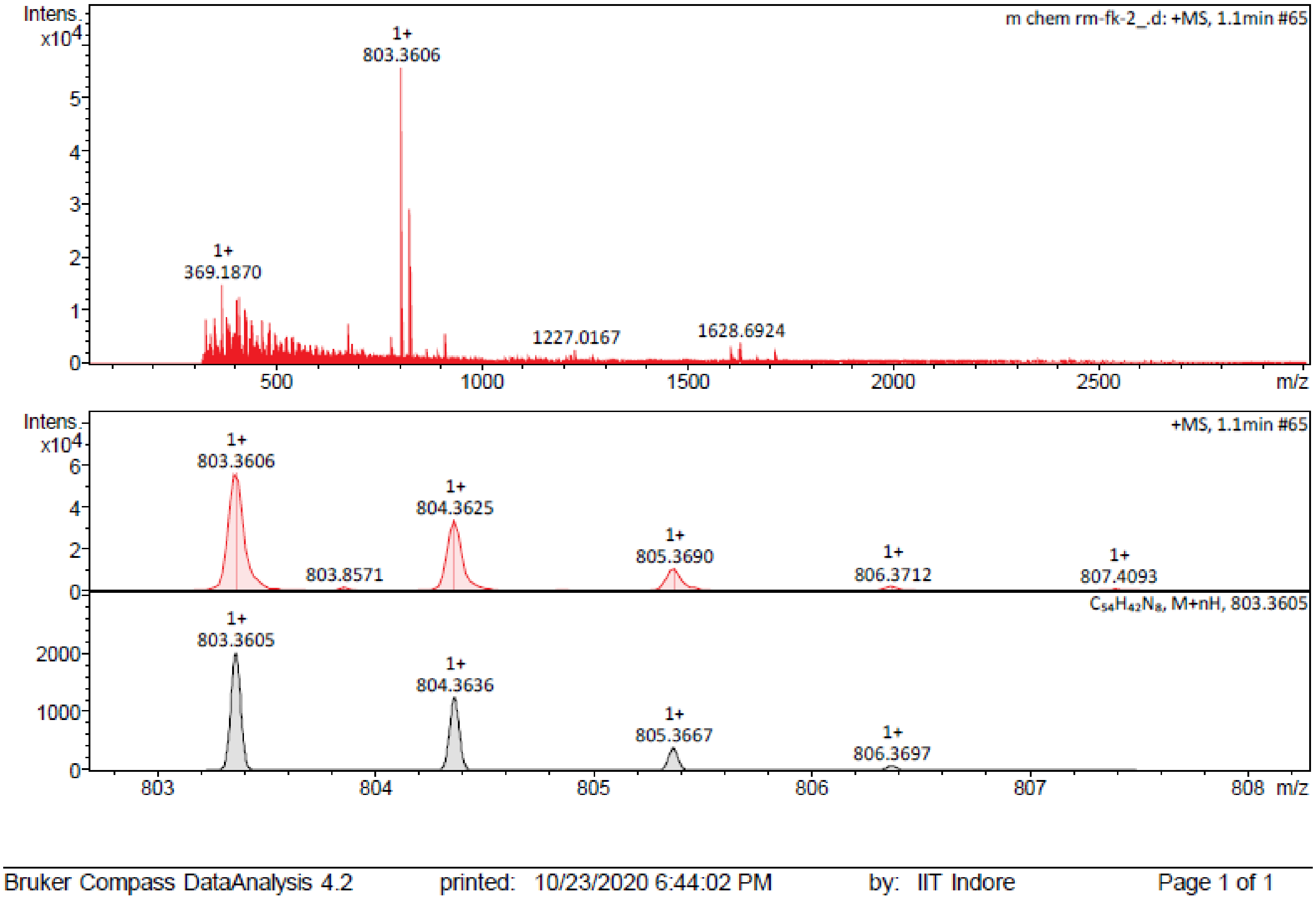

Figure S6. HRMS Spectra of **(NND-TCBD$_1$)$_3$-TPA, 2.**

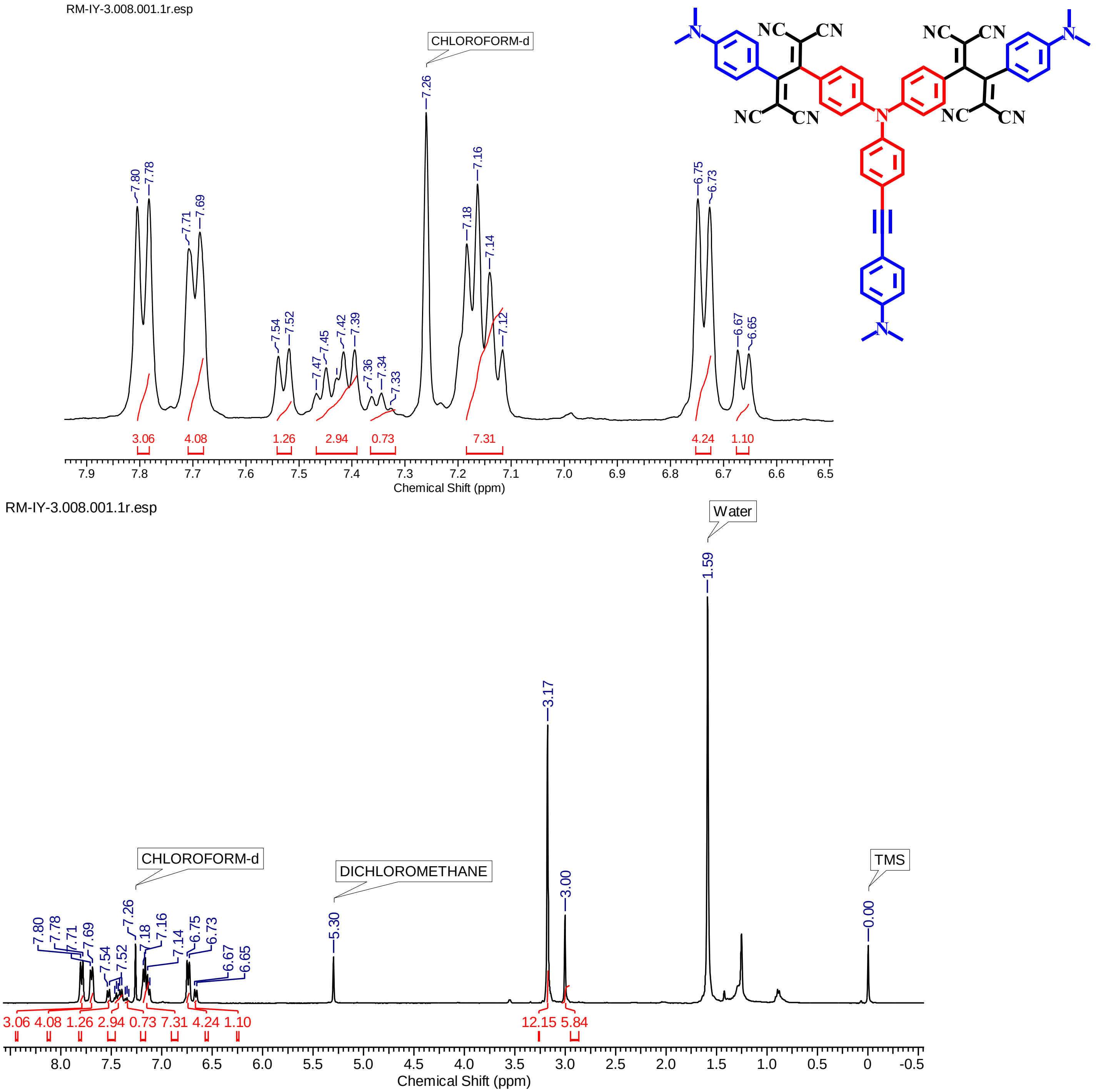

Figure S7. ^{1}H NMR Spectra of **(NND-TCBD$_2$)$_3$-TPA, 3.**

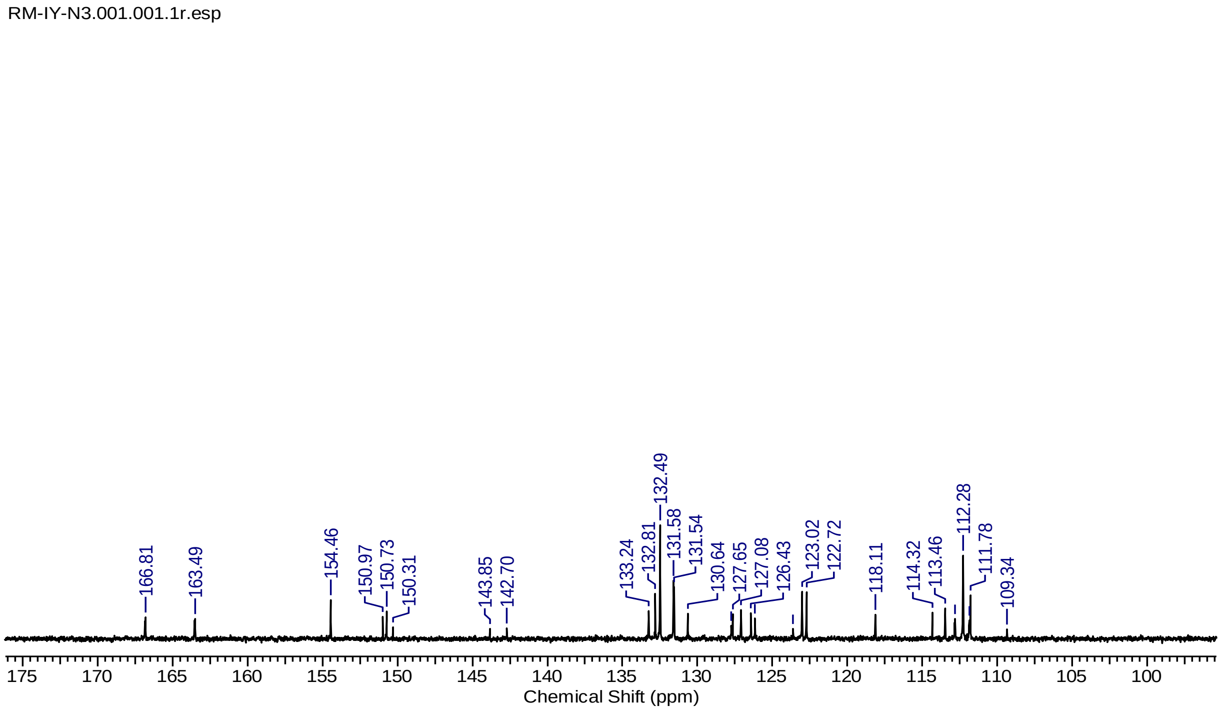

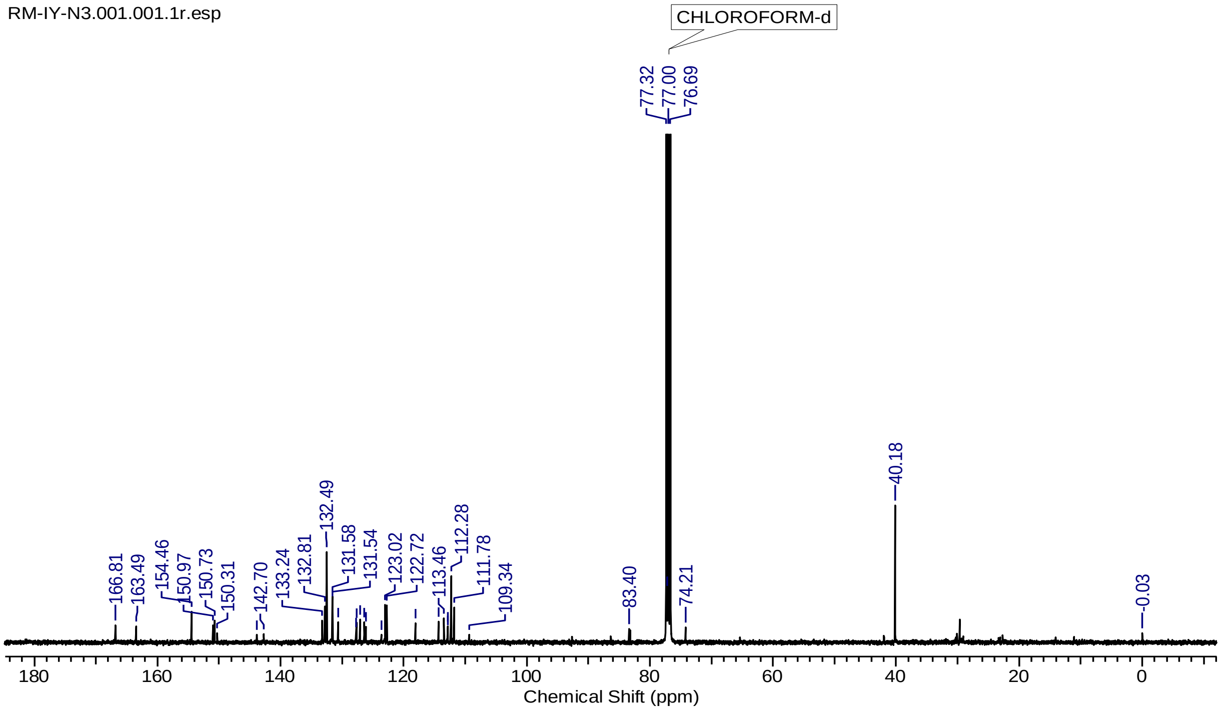

Figure S8. ^{13}C NMR Spectra of **(NND-TCBD$_2$)$_3$-TPA, 3.**

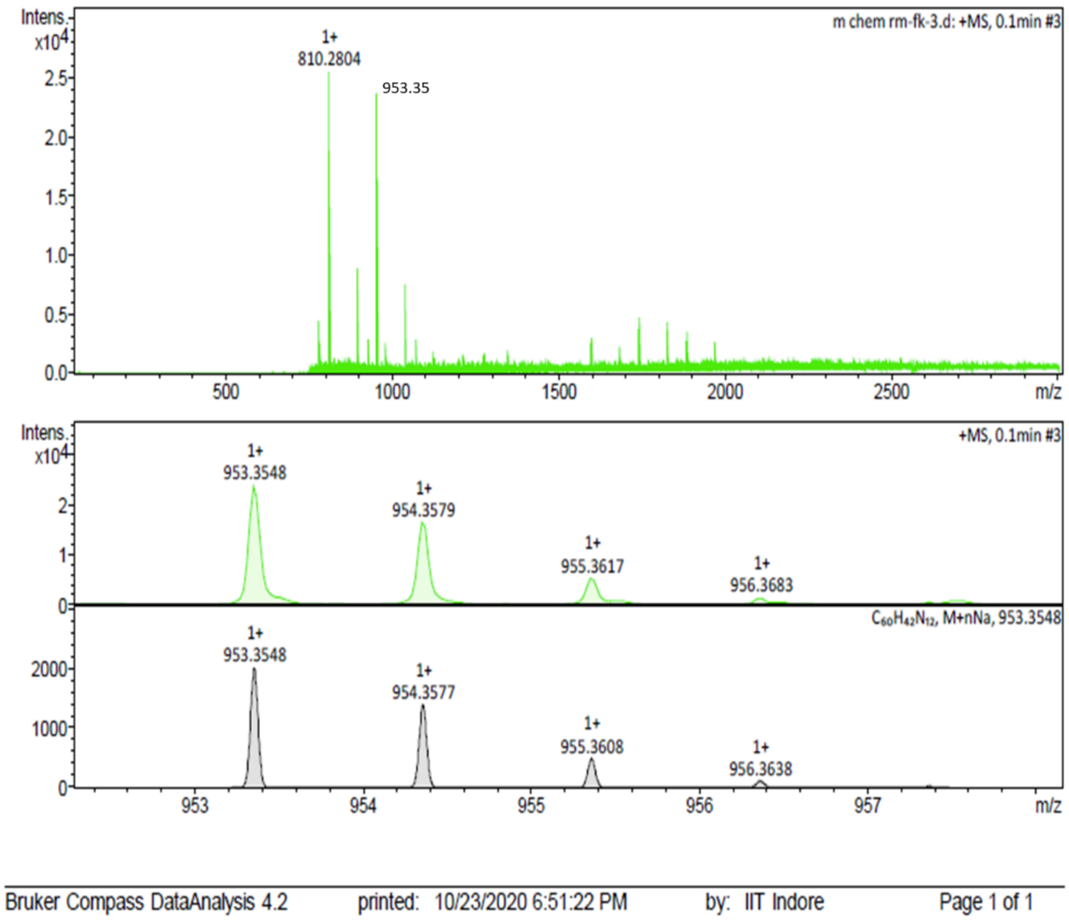

Figure S9. HRMS Spectra of **(NND-TCBD$_2$)$_3$-TPA, 3.**

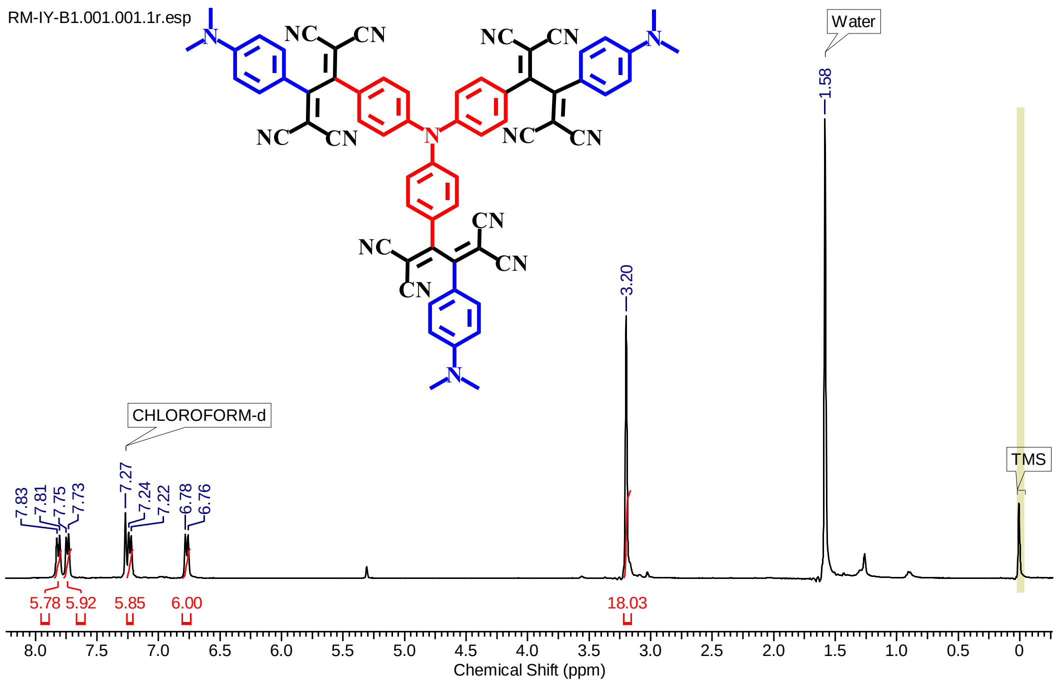

Figure S10. ^{1}H NMR Spectra of **(NND-TCBD$_3$)$_3$-TPA, 4.**

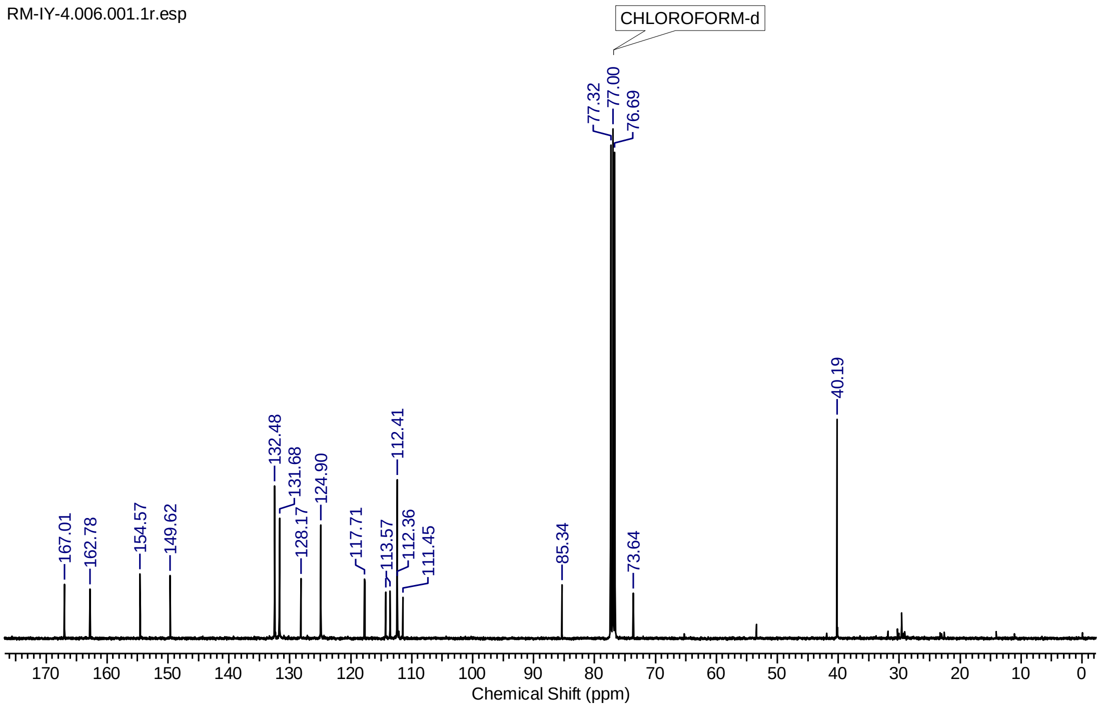

Figure S11. ^{13}C NMR Spectra of **(NND-TCBD$_3$)$_3$-TPA, 4.**

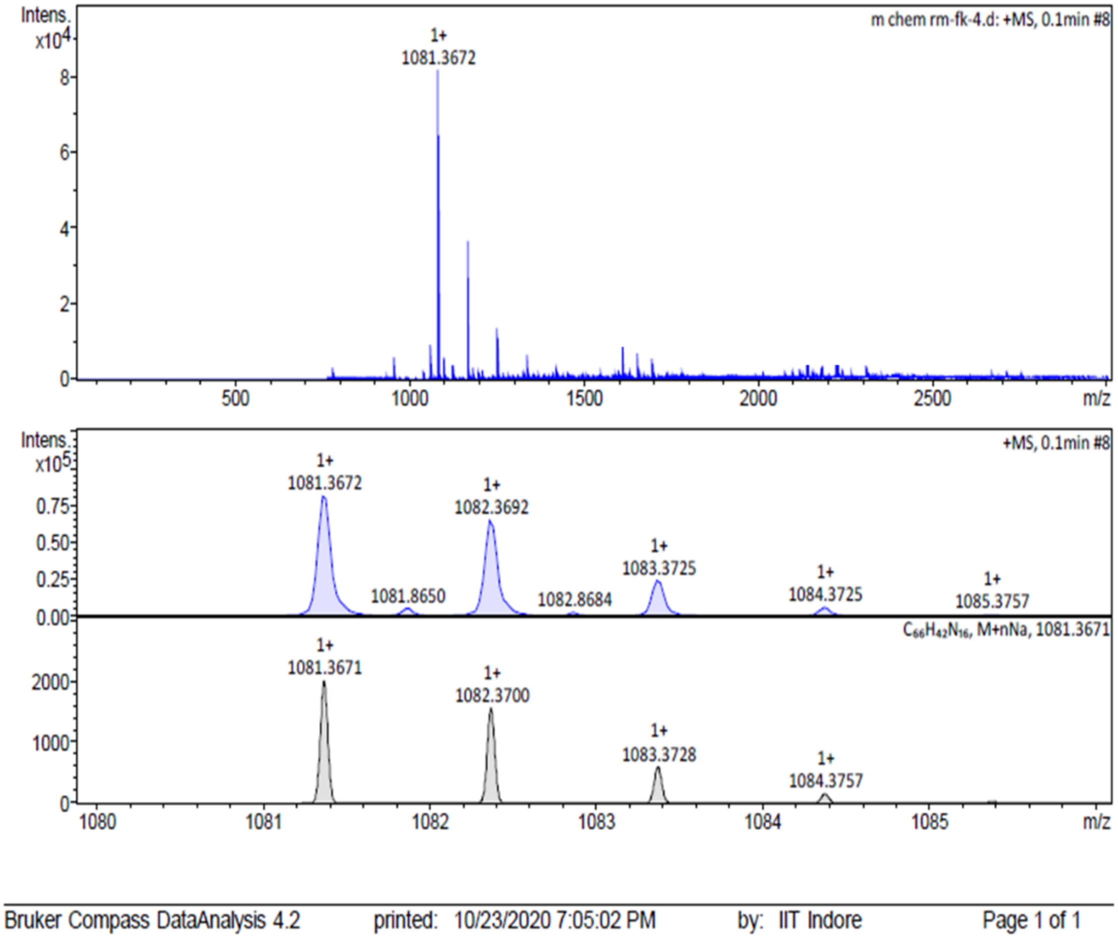

Figure S12. HRMS Spectra of **(NND-TCBD$_3$)$_3$-TPA, 4.**

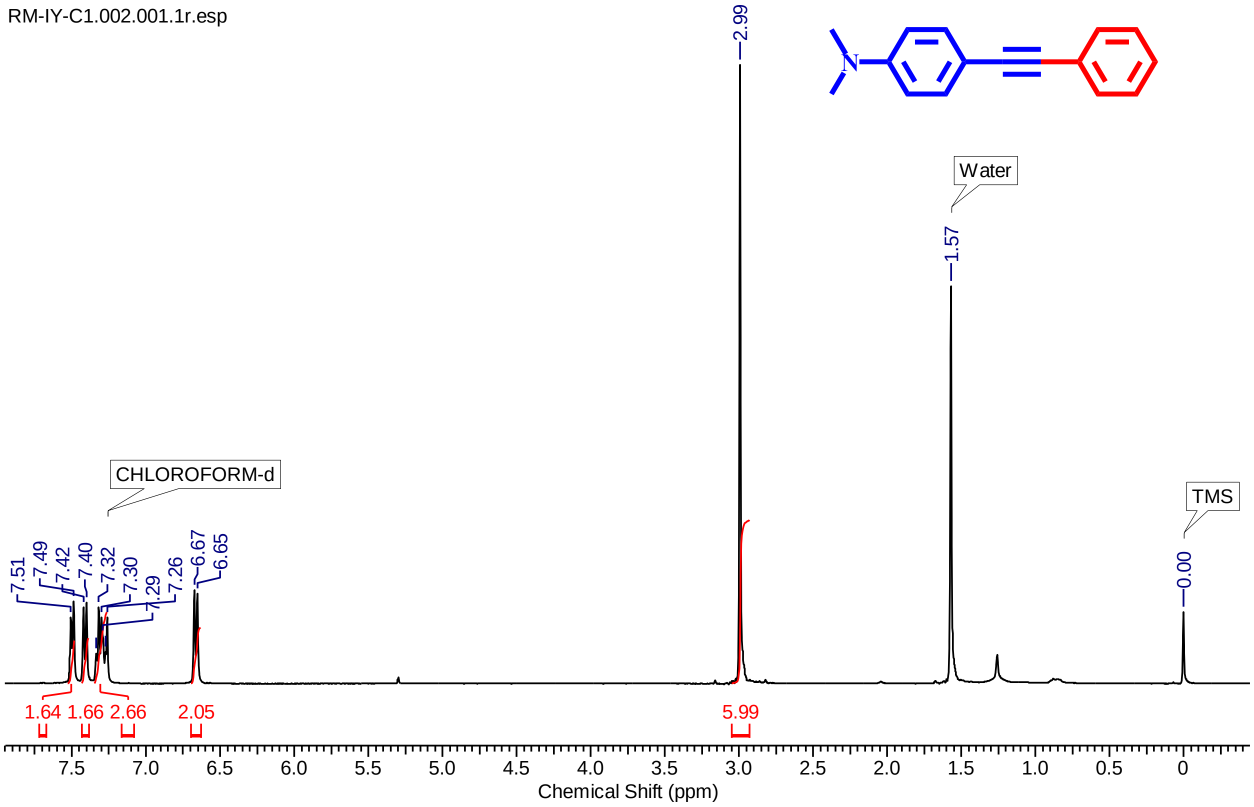

Figure S13. ^{1}H NMR Spectra of compound **C1.**

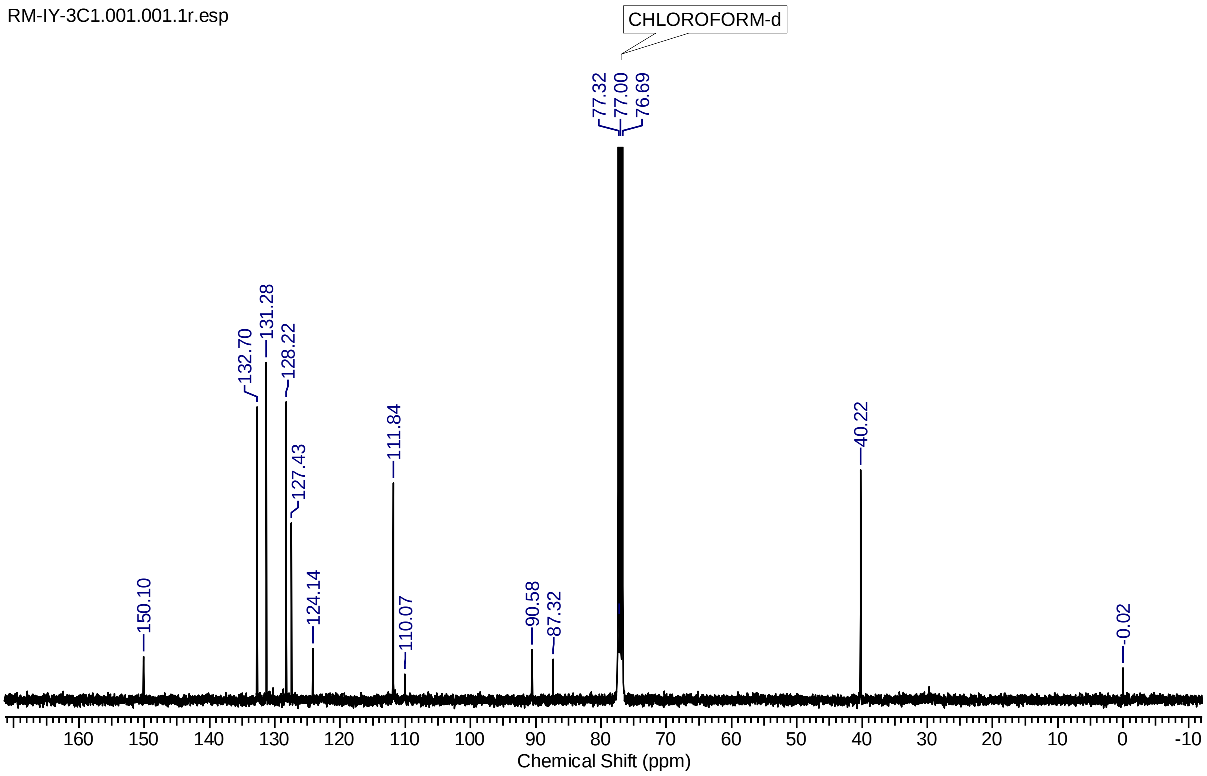

Figure S14. ^{13}C NMR Spectra of compound **C1.**

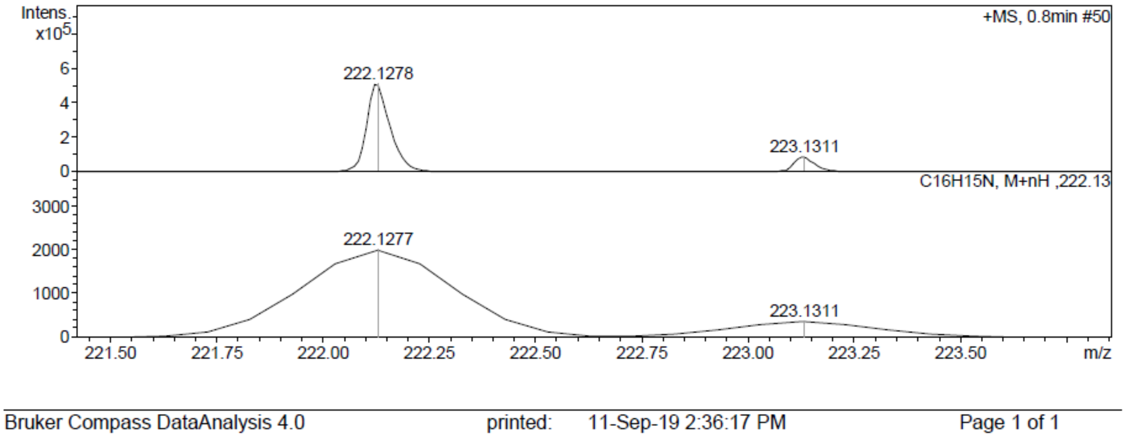

Figure S15. HRMS Spectra of compound **C1.**

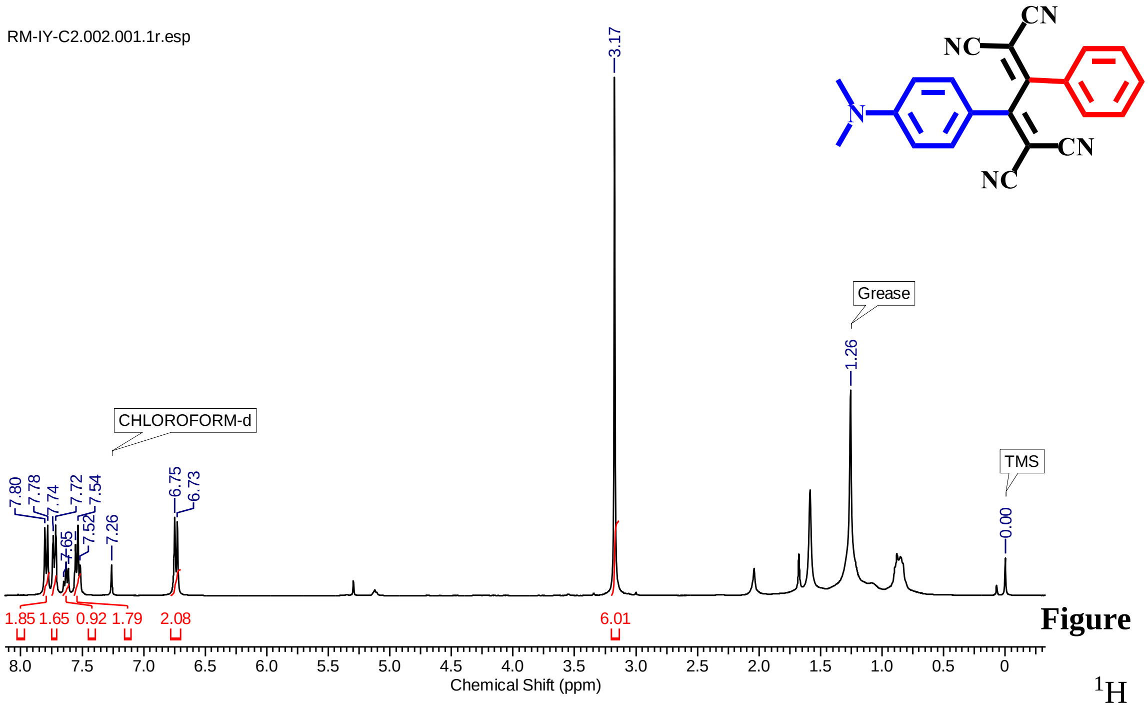

Figure S16. ^{1}H NMR Spectra of compound **C2.**

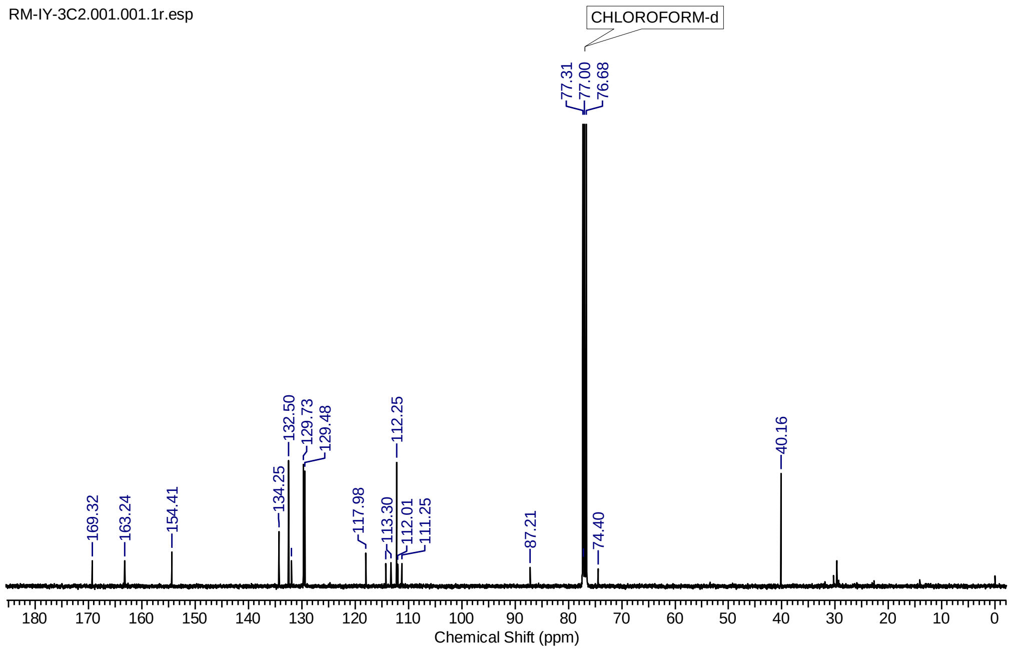

Figure S17. ^{13}C NMR Spectra of compound **C2.**

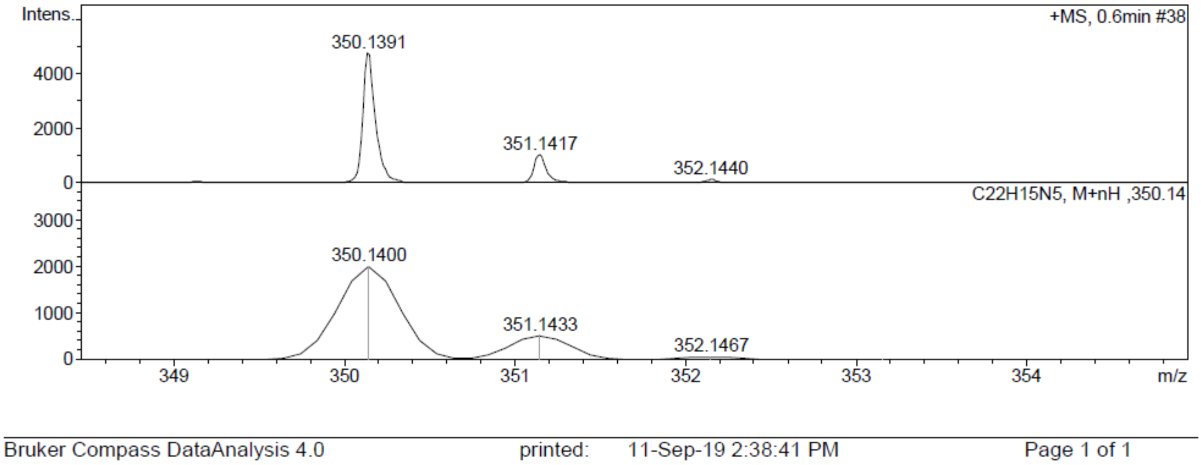

Figure S18. HRMS Spectra of compound **C2.**

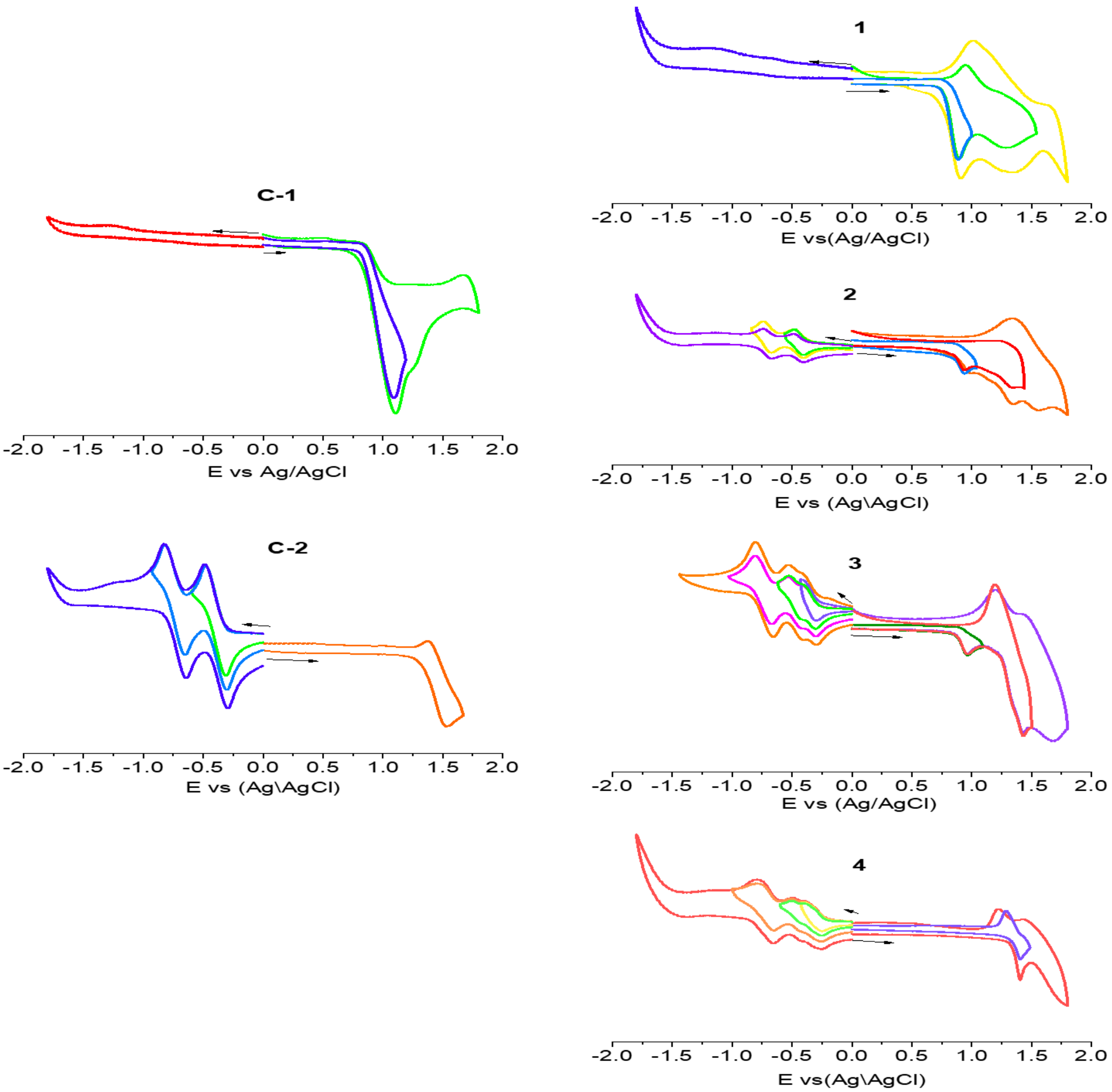

Figure S19. CVs of indicated compounds in DCB containing 0.1 M (TBA)ClO_4. Scan rate = 100 mV/s.

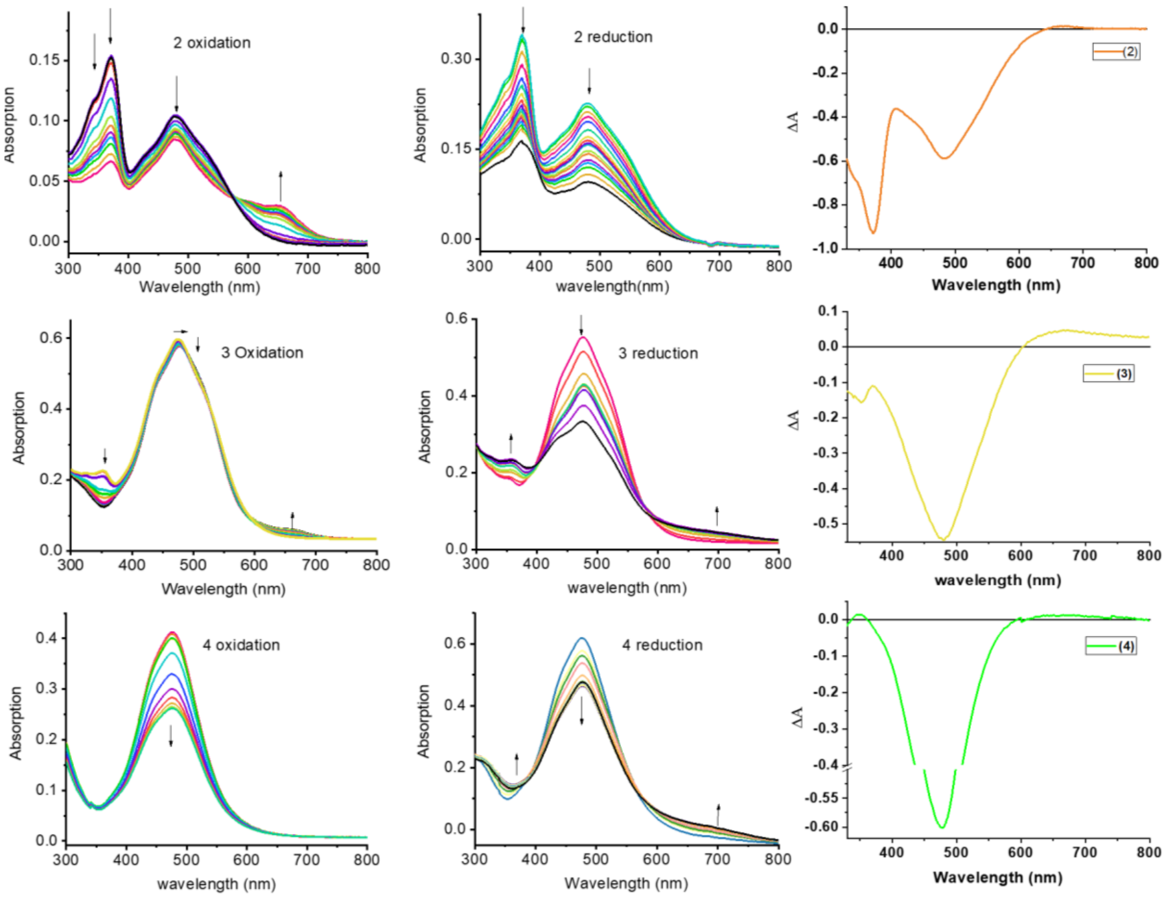

Figure S20. Spectral changes observed during (a) first oxidation and (b) first reduction of indicated compounds in DCB containing 0.2 M $(TBA)ClO_4$. Spectrum deduced for the charge transfer state using spectroelectrochemical data (see text for details) is show on the right hand panel for each compound.

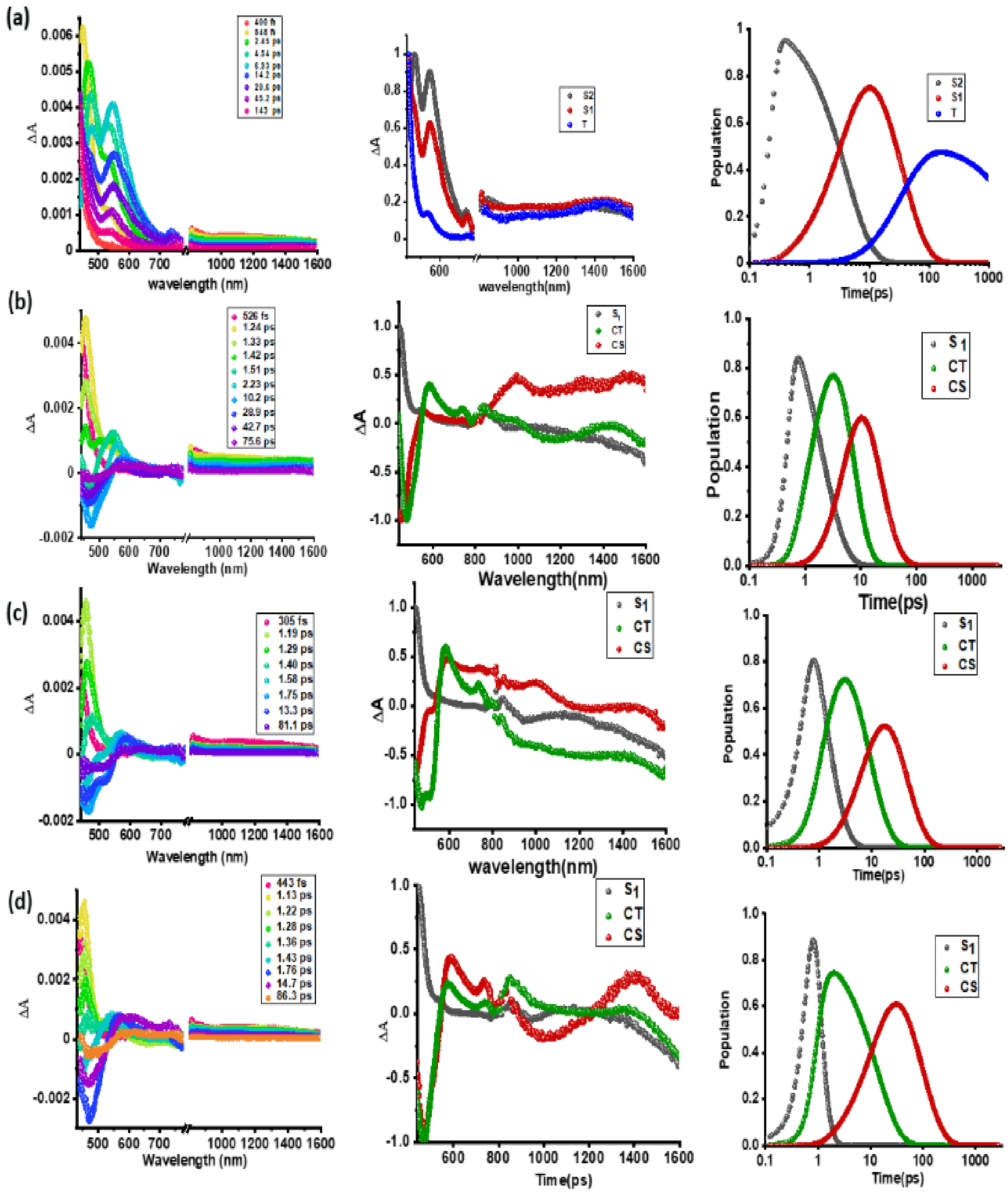

Figure S21. Fs-TA spectra at the indicated delay times of compounds **1-4** in DCB. The samples were excited at 350 nm corresponding to local excited state. The SAS and population kinetic plots are shown in the middle and right panels.

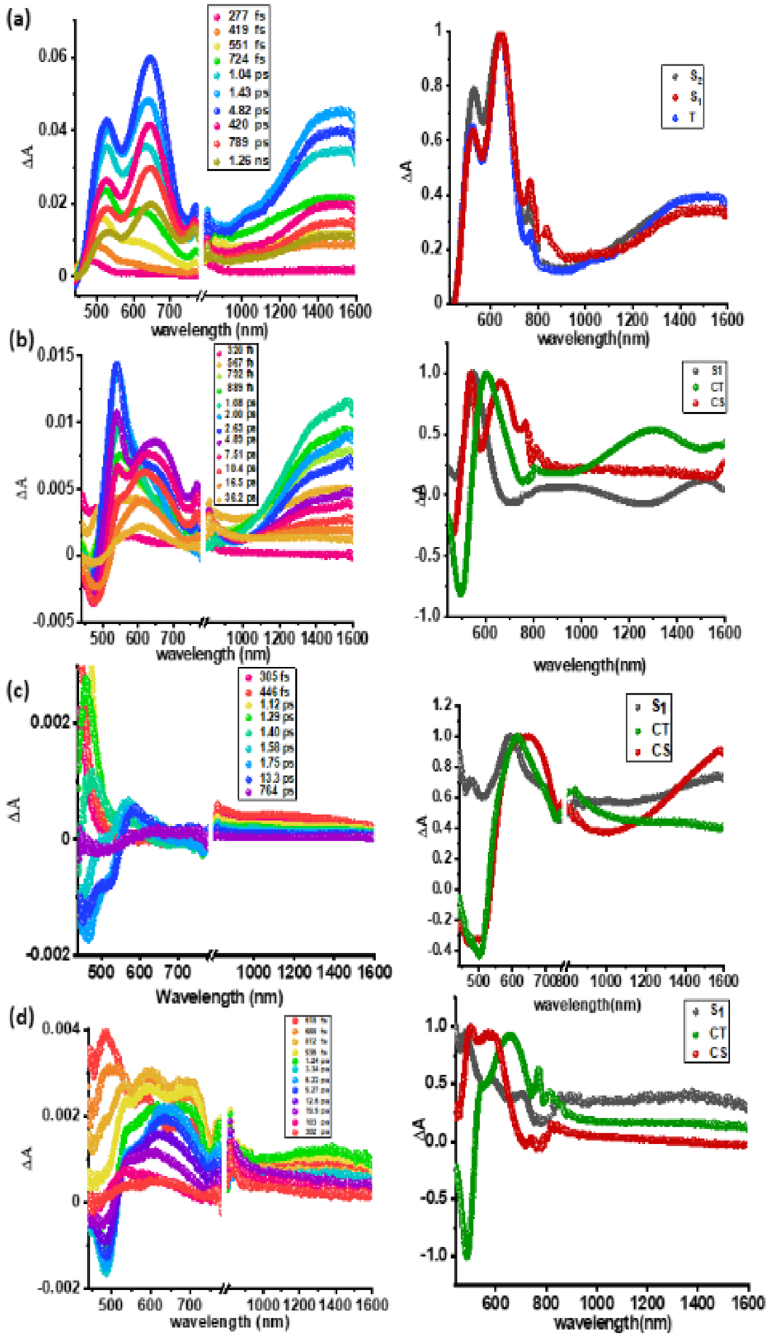

Figure S22. Fs-TA spectra at the indicated delay times of compounds **1-4** in toluene. The samples were excited at 350 nm corresponding to local excited state. The SAS and population kinetic plots are shown in the middle and right panels.

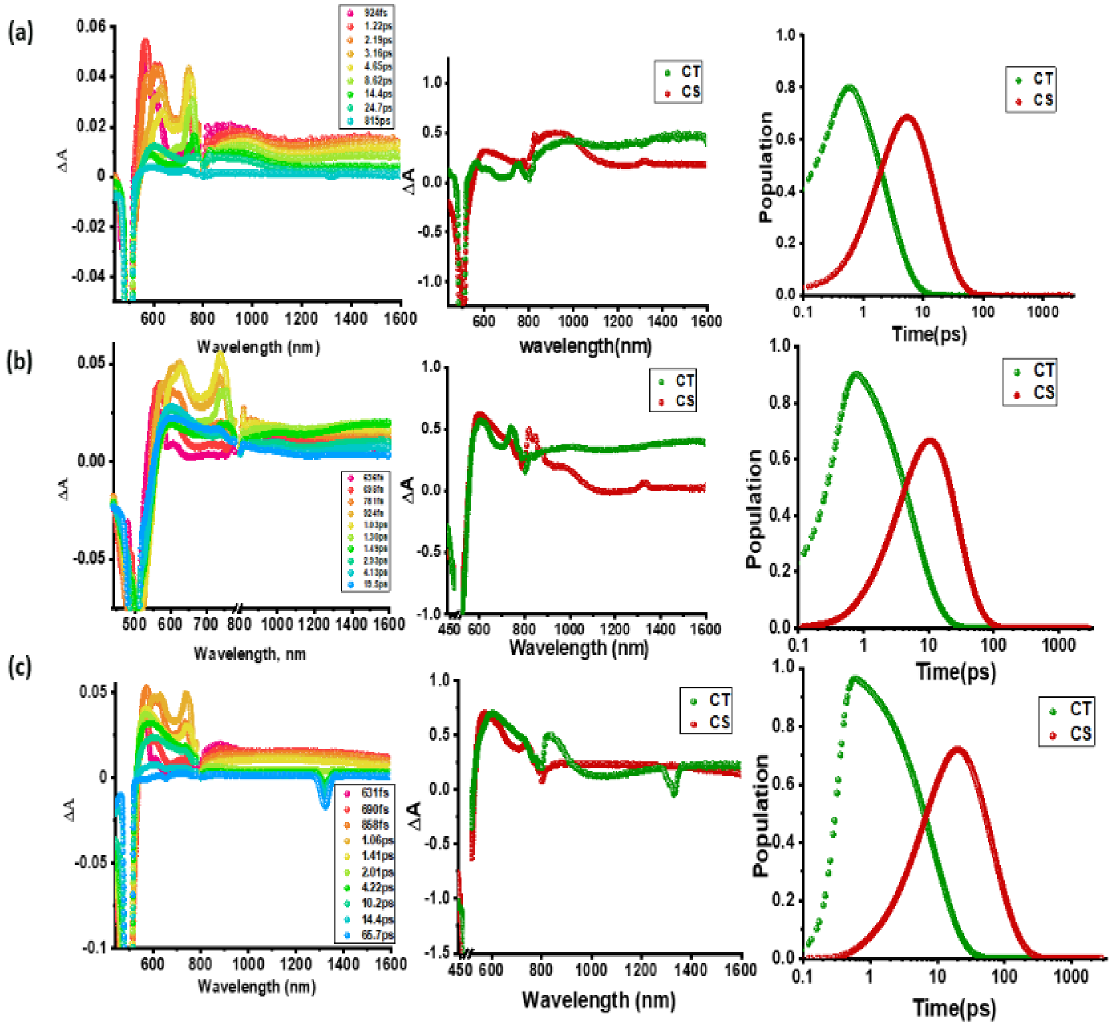

Figure S23. Fs-TA spectra at the indicated delay times of compounds **2-4** in DCB. The samples were excited at 500 nm corresponding to charge transfer band. Right hand panel shows the population kinetics. The dip at 500 nm is due to excitation laser.

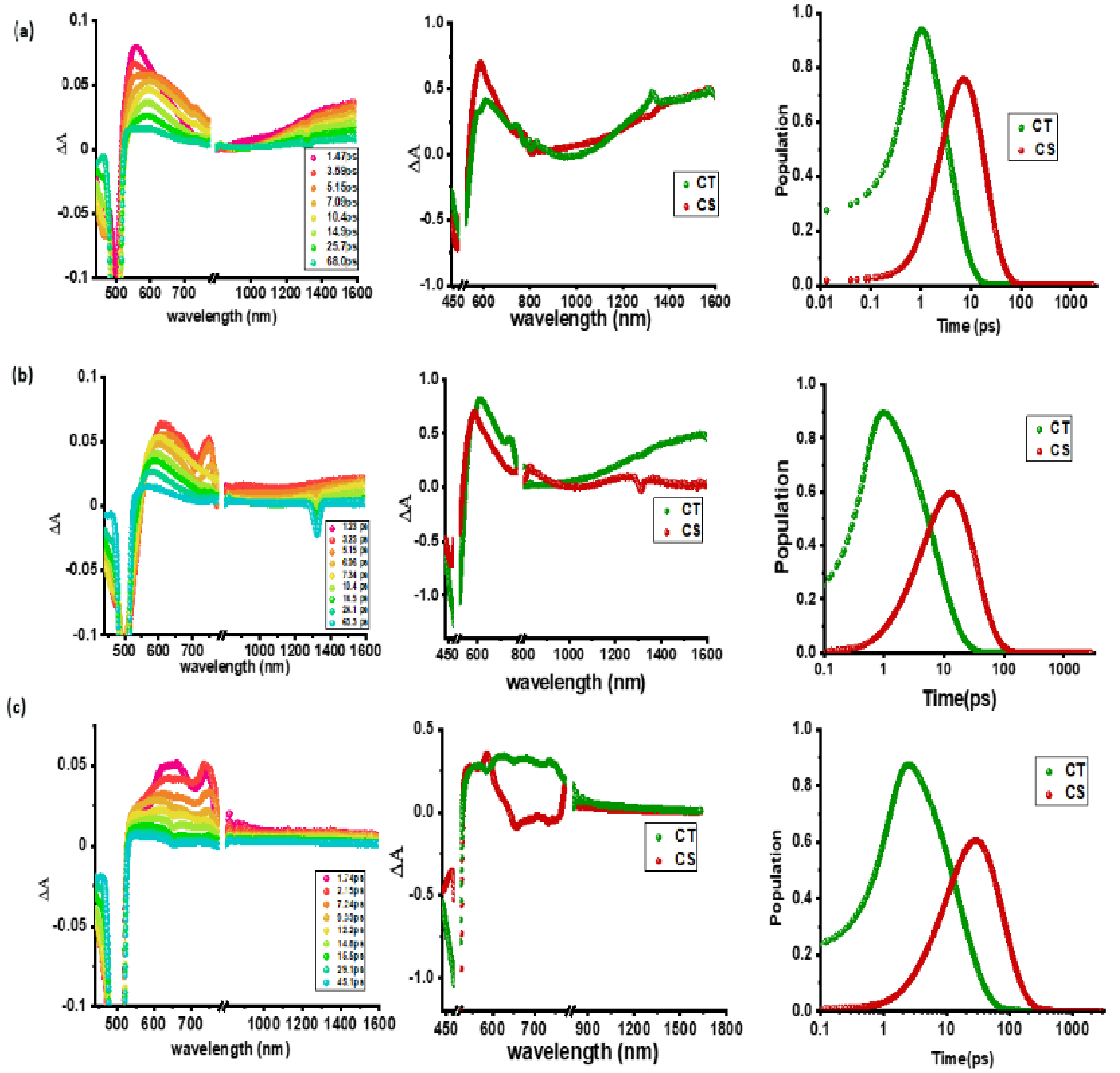

Figure S24. Fs-TA spectra at the indicated delay times of compounds **2-4** in toluene. The samples were excited at 500 nm corresponding to charge transfer band. Right hand panel shows the population kinetics. The dip at 500 nm is due to excitation laser.

DFT Calculation data

Calculation method: B3LYP/6-31+G for C, H and N with Gaussian 09.**

$(NND)_3$-TPA, 1:

Standard orientation:

Center Number	Atomic Number	Atomic Type	Coordinates (Angstroms) X	Y	Z
1	6	0	-0.457093	1.339345	0.008696
2	6	0	-1.552205	1.719764	-0.785958
3	6	0	0.172444	2.315800	0.799647
4	6	0	-2.007173	3.031055	-0.783041
5	1	0	-2.043060	0.978363	-1.407215
6	6	0	-0.273375	3.630230	0.789603
7	1	0	1.014270	2.034940	1.423356
8	6	0	-1.376506	4.019426	0.001319
9	1	0	-2.852013	3.309809	-1.404360
10	1	0	0.221795	4.372016	1.407706
11	6	0	1.398051	-0.274225	0.008890
12	6	0	2.274621	0.485361	-0.784954
13	6	0	1.929892	-1.307821	0.799106
14	6	0	3.637873	0.224459	-0.782669
15	1	0	1.877542	1.281470	-1.405496
16	6	0	3.291243	-1.578195	0.788280
17	1	0	1.266473	-1.897389	1.422649
18	6	0	4.179288	-0.816491	0.000289
19	1	0	4.301104	0.817639	-1.403771
20	1	0	3.686645	-2.378265	1.405568
21	6	0	5.574418	-1.089808	-0.005708
22	6	0	-1.837346	5.364312	-0.003638
23	6	0	6.769658	-1.324523	-0.012098
24	6	0	-2.232571	6.516481	-0.009505
25	6	0	8.164301	-1.598617	-0.021585
26	6	0	9.060180	-0.816224	-0.776764
27	6	0	8.708362	-2.660275	0.727963
28	6	0	10.421603	-1.078956	-0.788928
29	1	0	8.673135	0.014126	-1.358958
30	6	0	10.068280	-2.931155	0.722214
31	1	0	8.045324	-3.277021	1.326495
32	6	0	10.967025	-2.153474	-0.046423
33	1	0	11.065007	-0.441157	-1.381929
34	6	0	-2.694034	7.860787	-0.019159
35	6	0	-2.061620	8.859208	0.747818
36	6	0	-3.806403	8.248410	-0.792065
37	6	0	-2.508931	10.171704	0.742215

38	1	0	-1.206345	8.591036	1.360046
39	6	0	-4.261338	9.558169	-0.804293
40	1	0	-4.320247	7.500907	-1.388248
41	6	0	-3.618603	10.564190	-0.043881
42	1	0	-1.989442	10.896725	1.356154
43	7	0	12.323755	-2.437510	-0.077567
44	7	0	-4.052299	11.880646	-0.074796
45	6	0	-0.927423	-1.074239	0.013675
46	6	0	-2.090176	-1.012004	0.800987
47	6	0	-0.706842	-2.218872	-0.771723
48	6	0	-3.004975	-2.055830	0.796312
49	1	0	-2.270094	-0.138176	1.417891
50	6	0	-1.614275	-3.269099	-0.762934
51	1	0	0.182280	-2.277850	-1.390188
52	6	0	-2.787649	-3.211924	0.017853
53	1	0	-3.896410	-1.993794	1.411936
54	1	0	-1.430838	-4.144816	-1.376921
55	7	0	0.004449	-0.003372	0.011892
56	6	0	-3.720403	-4.284811	0.020288
57	6	0	-4.519011	-5.204580	0.022484
58	6	0	-5.449407	-6.279120	0.025463
59	6	0	-5.209111	-7.459008	-0.706101
60	6	0	-6.652612	-6.205667	0.754837
61	6	0	-6.114137	-8.509550	-0.707797
62	1	0	-4.294664	-7.543378	-1.284803
63	6	0	-7.564136	-7.250568	0.759204
64	1	0	-6.870885	-5.306565	1.322468
65	6	0	-7.317170	-8.441227	0.034795
66	1	0	-8.476376	-7.137640	1.331481
67	7	0	-8.212552	-9.499277	0.057988
68	1	0	-5.124798	9.798760	-1.411670
69	1	0	-5.882060	-9.389992	-1.293773
70	1	0	10.432927	-3.755378	1.322309
71	6	0	-5.298871	12.210305	-0.744184
72	1	0	-5.260491	11.941323	-1.806100
73	1	0	-6.169759	11.705601	-0.298415
74	1	0	-5.462663	13.287113	-0.684711
75	6	0	-3.486204	12.845049	0.852590
76	1	0	-3.669505	12.583568	1.906179
77	1	0	-2.403307	12.939573	0.712052
78	1	0	-3.927618	13.824445	0.663426
79	6	0	-9.514959	-9.324050	0.677182
80	1	0	-9.417105	-9.066550	1.738190
81	1	0	-10.116062	-8.539531	0.192387
82	1	0	-10.068389	-10.262128	0.617362
83	6	0	-8.013249	-10.628374	-0.834061
84	1	0	-8.044863	-10.344230	-1.897167
85	1	0	-7.050491	-11.116765	-0.644202
86	1	0	-8.795898	-11.367056	-0.656085
87	6	0	12.870693	-3.426727	0.835111
88	1	0	12.731854	-3.155504	1.893041
89	1	0	12.410028	-4.408414	0.675541

90	1	0	13.940197	-3.532656	0.648575
91	6	0	13.236322	-1.508992	-0.722431
92	1	0	13.233662	-0.512497	-0.254504
93	1	0	14.250099	-1.907853	-0.667529
94	1	0	12.988913	-1.384115	-1.782891

Total Energy (HF) = -2073.3075124 Hartrees

$(NND-TCBD_1)_3$-TPA, 2:

Standard orientation:

Center Number	Atomic Number	Atomic Type	Coordinates (Angstroms) X	Y	Z
1	6	0	2.097750	-0.087951	-0.225210
2	6	0	2.972511	0.734966	-0.950004
3	6	0	2.630191	-1.075891	0.617391
4	6	0	4.346618	0.572698	-0.837035
5	1	0	2.567249	1.500987	-1.602513
6	6	0	4.004195	-1.248437	0.718678
7	1	0	1.961114	-1.707584	1.192272
8	6	0	4.894088	-0.426064	-0.004174
9	1	0	5.014732	1.211687	-1.404611
10	1	0	4.405746	-2.015657	1.372070
11	6	0	-0.175689	-0.980835	-0.518292
12	6	0	0.267436	-2.161560	-1.156713
13	6	0	-1.521794	-0.923813	-0.093781
14	6	0	-0.584196	-3.232774	-1.343569
15	1	0	1.281920	-2.217435	-1.532426
16	6	0	-2.371692	-1.993619	-0.300294
17	1	0	-1.890875	-0.035200	0.403735
18	6	0	-1.930966	-3.191356	-0.912168
19	1	0	-0.210829	-4.095174	-1.880072
20	1	0	-3.395635	-1.914861	0.049055
21	6	0	6.300279	-0.597626	0.104418
22	6	0	7.505979	-0.745549	0.195096
23	6	0	-6.543965	-4.534009	-0.056348
24	6	0	-4.534166	-4.515140	1.269121
25	6	0	-7.279599	-4.875672	1.060063
26	1	0	-7.062981	-4.462305	-1.002923
27	6	0	-5.260397	-4.841137	2.397397
28	1	0	-3.462158	-4.383171	1.370229
29	6	0	-6.665544	-5.028632	2.331321
30	1	0	-8.341671	-5.046530	0.941872
31	6	0	8.911726	-0.919696	0.301103
32	6	0	9.465034	-1.933815	1.108075

33	6	0	9.807986	-0.090182	-0.401989
34	6	0	10.835711	-2.109810	1.213193
35	1	0	8.799869	-2.594228	1.655380
36	6	0	11.180192	-0.258346	-0.304027
37	1	0	9.411776	0.695011	-1.038106
38	6	0	11.737597	-1.269469	0.516079
39	1	0	11.207267	-2.909320	1.841591
40	7	0	-7.397097	-5.353595	3.443470
41	7	0	13.107264	-1.425502	0.635896
42	6	0	0.178087	1.434525	-0.239802
43	6	0	0.593366	2.279903	0.799297
44	6	0	-0.717473	1.923045	-1.203185
45	6	0	0.122264	3.583829	0.875767
46	1	0	1.287980	1.907206	1.544911
47	6	0	-1.199677	3.222517	-1.119047
48	1	0	-1.030466	1.279492	-2.018878
49	6	0	-0.788699	4.083284	-0.079016
50	1	0	0.447033	4.229929	1.684469
51	1	0	-1.890175	3.592054	-1.869678
52	7	0	0.685569	0.101172	-0.326462
53	6	0	-1.276657	5.415322	0.001429
54	6	0	-1.696997	6.556719	0.067144
55	6	0	-2.189702	7.886933	0.141172
56	6	0	-3.126144	8.373290	-0.792888
57	6	0	-1.757870	8.774146	1.147204
58	6	0	-3.609232	9.670501	-0.726816
59	1	0	-3.472966	7.715600	-1.583628
60	6	0	-2.234617	10.073421	1.221765
61	1	0	-1.031661	8.430960	1.877313
62	6	0	-3.183840	10.559843	0.290000
63	1	0	-1.863932	10.715147	2.011196
64	7	0	-3.679823	11.848452	0.372843
65	6	0	-5.145556	-4.329084	0.006297
66	6	0	-2.861287	-4.297518	-1.095355
67	6	0	-4.334665	-3.970690	-1.148147
68	6	0	-4.778902	-3.340214	-2.294583
69	6	0	-2.521241	-5.630998	-1.211557
70	6	0	-3.512239	-6.628959	-1.485361
71	6	0	-1.199172	-6.150733	-1.040858
72	6	0	-6.101778	-2.830094	-2.483481
73	6	0	-3.902408	-3.118520	-3.405884
74	7	0	-7.160076	-2.381618	-2.671890
75	7	0	-3.212788	-2.926988	-4.324325
76	7	0	-4.296027	-7.460188	-1.709838
77	7	0	-0.145983	-6.622610	-0.884154
78	1	0	-4.734703	-4.957956	3.336199
79	1	0	-4.322980	9.994534	-1.473655
80	1	0	11.823679	0.401646	-0.871936
81	6	0	13.997962	-0.655353	-0.215703
82	1	0	13.848207	-0.864609	-1.285528
83	1	0	13.863159	0.421455	-0.059883
84	1	0	15.031197	-0.896847	0.036248

85	6	0	13.642978	-2.566311	1.359265
86	1	0	13.301144	-2.570942	2.400837
87	1	0	13.360711	-3.528310	0.905586
88	1	0	14.731667	-2.502981	1.371152
89	6	0	-3.114285	12.779406	1.334356
90	1	0	-3.227705	12.408836	2.360030
91	1	0	-2.045031	12.971716	1.159889
92	1	0	-3.645186	13.729655	1.267536
93	6	0	-4.536209	12.362249	-0.682758
94	1	0	-4.033958	12.387922	-1.661384
95	1	0	-5.445666	11.758568	-0.785888
96	1	0	-4.842647	13.378314	-0.431892
97	6	0	-8.832905	-5.577585	3.338925
98	1	0	-9.345048	-4.697244	2.933736
99	1	0	-9.070447	-6.437961	2.699447
100	1	0	-9.236851	-5.771643	4.332281
101	6	0	-6.730018	-5.586966	4.716832
102	1	0	-6.017926	-6.420435	4.659239
103	1	0	-6.187871	-4.695805	5.055872
104	1	0	-7.476572	-5.831532	5.472007

Total Energy (HF) = -2520.9021615 Hartrees

(NND-TCBD$_2$)$_3$-TPA, 3:

Standard orientation:

Center Number	Atomic Number	Atomic Type	Coordinates (Angstroms) X	Y	Z
1	6	0	1.272301	-1.466049	-0.489043
2	6	0	2.338140	-1.409339	-1.397443
3	6	0	1.339923	-2.344401	0.600980
4	6	0	3.457045	-2.211131	-1.213800
5	1	0	2.283755	-0.735702	-2.246482
6	6	0	2.452264	-3.156994	0.777349
7	1	0	0.516913	-2.386753	1.307070
8	6	0	3.537378	-3.104594	-0.123825
9	1	0	4.277987	-2.164569	-1.921070
10	1	0	2.499616	-3.835011	1.622631
11	6	0	-1.156204	-1.213472	-0.714479
12	6	0	-1.321501	-2.519248	-1.217400
13	6	0	-2.293631	-0.527361	-0.244594
14	6	0	-2.567653	-3.120230	-1.233426
15	1	0	-0.466335	-3.051248	-1.616600
16	6	0	-3.542166	-1.124485	-0.288779
17	1	0	-2.193939	0.472979	0.159640

18	6	0	-3.712848	-2.445093	-0.759153
19	1	0	-2.658951	-4.107077	-1.668667
20	1	0	-4.397862	-0.570253	0.082130
21	6	0	4.679913	-3.927146	0.061572
22	6	0	5.661883	-4.630555	0.219971
23	6	0	-8.423631	-1.480924	0.152986
24	6	0	-6.566008	-2.137074	1.537604
25	6	0	-9.193987	-1.278148	1.279285
26	1	0	-8.890491	-1.338965	-0.812425
27	6	0	-7.320229	-1.921722	2.673737
28	1	0	-5.543734	-2.479130	1.659676
29	6	0	-8.665838	-1.479036	2.582455
30	1	0	-10.225061	-0.976896	1.148385
31	6	0	6.807962	-5.448108	0.403268
32	6	0	6.862796	-6.413151	1.429138
33	6	0	7.936712	-5.322460	-0.431713
34	6	0	7.980274	-7.210968	1.613684
35	1	0	6.009071	-6.531151	2.088962
36	6	0	9.059571	-6.114341	-0.255088
37	1	0	7.924280	-4.585696	-1.228616
38	6	0	9.113798	-7.088382	0.772461
39	1	0	7.973386	-7.935480	2.418121
40	7	0	-9.425871	-1.265671	3.701234
41	7	0	10.225647	-7.888089	0.945428
42	6	0	0.316040	0.762488	-0.816766
43	6	0	1.364309	1.406084	-0.131429
44	6	0	-0.495508	1.529207	-1.676203
45	6	0	1.582877	2.763171	-0.296697
46	1	0	2.005129	0.832838	0.528024
47	6	0	-0.287891	2.890444	-1.817465
48	1	0	-1.270826	1.045551	-2.258894
49	6	0	0.749636	3.548814	-1.122755
50	1	0	2.396641	3.231011	0.247421
51	1	0	-0.903222	3.435791	-2.521698
52	7	0	0.123183	-0.626428	-0.672551
53	6	0	2.758272	6.235000	0.080651
54	6	0	1.966051	6.118276	1.247839
55	6	0	3.842317	7.142546	0.138395
56	6	0	2.250022	6.819942	2.402488
57	1	0	1.114407	5.446042	1.252957
58	6	0	4.126443	7.863764	1.279503
59	1	0	4.452297	7.317621	-0.737879
60	6	0	3.347522	7.718626	2.458415
61	1	0	4.950937	8.564001	1.252764
62	7	0	3.636801	8.424378	3.595617
63	6	0	-7.076800	-1.907503	0.237352
64	6	0	-5.046196	-3.049564	-0.764550
65	6	0	-6.233394	-2.132574	-0.926983
66	6	0	-6.400039	-1.576676	-2.181667
67	6	0	-5.303012	-4.394116	-0.620086
68	6	0	-6.633651	-4.917371	-0.727824
69	6	0	-4.306274	-5.377536	-0.317814

70	6	0	-7.380469	-0.590156	-2.513841
71	6	0	-5.553819	-1.955931	-3.273346
72	7	0	-8.152839	0.224897	-2.822870
73	7	0	-4.876565	-2.255414	-4.171970
74	7	0	-7.701599	-5.372067	-0.812633
75	7	0	-3.535622	-6.206212	-0.044753
76	6	0	1.004954	4.984392	-1.258204
77	6	0	0.038581	5.935018	-1.497402
78	6	0	2.427157	5.462229	-1.107301
79	6	0	3.302354	5.104217	-2.115729
80	6	0	0.378155	7.310967	-1.714892
81	6	0	-1.367916	5.664075	-1.513946
82	7	0	0.623710	8.433529	-1.899247
83	7	0	-2.519564	5.494663	-1.504505
84	6	0	2.841828	4.428661	-3.291655
85	6	0	4.713373	5.335519	-2.086615
86	7	0	2.487971	3.880491	-4.256102
87	7	0	5.868458	5.484465	-2.094592
88	1	0	1.613240	6.678418	3.265953
89	1	0	-6.867499	-2.105085	3.639453
90	1	0	9.901653	-5.975935	-0.921219
91	6	0	10.283768	-8.822113	2.056814
92	1	0	10.225479	-8.316388	3.031416
93	1	0	9.469926	-9.556520	2.008203
94	1	0	11.226126	-9.368971	2.016443
95	6	0	11.406281	-7.679988	0.124214
96	1	0	11.185751	-7.820697	-0.941432
97	1	0	11.831615	-6.674240	0.251886
98	1	0	12.169509	-8.406806	0.403401
99	6	0	-8.878097	-1.538592	5.022909
100	1	0	-8.600705	-2.594078	5.140647
101	1	0	-7.990917	-0.925952	5.224593
102	1	0	-9.627790	-1.300045	5.776725
103	6	0	-10.816778	-0.848127	3.576481
104	1	0	-11.424195	-1.598663	3.054501
105	1	0	-11.236975	-0.705372	4.571643
106	1	0	-10.902396	0.100874	3.034395
107	6	0	4.745586	9.370329	3.611317
108	1	0	5.694308	8.877467	3.369220
109	1	0	4.593375	10.192339	2.899929
110	1	0	4.835375	9.798267	4.609393
111	6	0	2.780788	8.308522	4.768532
112	1	0	1.752993	8.631398	4.558198
113	1	0	2.747458	7.277456	5.141088
114	1	0	3.177275	8.939046	5.563797

Total Energy (HF) = -2968.4913852 Hartrees

(NND-TCBD$_3$)$_3$-TPA, 4:

Standard orientation:

Center Number	Atomic Number	Atomic Type	Coordinates (Angstroms) X	Y	Z
1	6	0	0.466000	-1.615736	0.098047
2	6	0	1.685801	-1.682791	-0.594635
3	6	0	0.237739	-2.494040	1.169074
4	6	0	2.657628	-2.593072	-0.208266
5	1	0	1.861658	-1.028175	-1.441042
6	6	0	1.217564	-3.395232	1.559524
7	1	0	-0.709814	-2.468526	1.695344
8	6	0	2.458588	-3.449506	0.894171
9	1	0	3.587123	-2.637027	-0.765304
10	1	0	1.004940	-4.073687	2.375728
11	6	0	-1.876290	-1.049089	-0.403606
12	6	0	-2.222462	-2.334231	-0.858228
13	6	0	-2.903936	-0.146314	-0.077505
14	6	0	-3.551840	-2.708312	-0.969361
15	1	0	-1.446065	-3.032046	-1.150127
16	6	0	-4.231690	-0.517868	-0.215158
17	1	0	-2.657782	0.846050	0.282333
18	6	0	-4.592075	-1.813203	-0.643337
19	1	0	-3.779357	-3.689485	-1.365617
20	1	0	-5.004109	0.199201	0.041343
21	6	0	-9.118123	0.029544	-0.283718
22	6	0	-7.583745	-0.904754	1.320258
23	6	0	-9.958859	0.412057	0.740600
24	1	0	-9.438599	0.219481	-1.299231
25	6	0	-8.407988	-0.513494	2.355956
26	1	0	-6.663884	-1.423626	1.568338
27	6	0	-9.629322	0.163694	2.100102
28	1	0	-10.894273	0.892001	0.484407
29	6	0	5.776036	-4.409940	0.113355
30	6	0	6.826037	-5.084935	-0.556319
31	6	0	6.106944	-3.171699	0.717693
32	6	0	8.095807	-4.553972	-0.639561
33	1	0	6.655785	-6.058983	-0.994605
34	6	0	7.368702	-2.620746	0.627923
35	1	0	5.347386	-2.616851	1.257951
36	6	0	8.410593	-3.292828	-0.064570
37	1	0	8.860064	-5.128424	-1.146380
38	7	0	-10.458797	0.550478	3.117556
39	7	0	9.663638	-2.755607	-0.165848
40	6	0	-0.111460	0.656978	-0.583151
41	6	0	0.856289	1.289850	0.216211
42	6	0	-0.636365	1.346951	-1.689830
43	6	0	1.297783	2.564771	-0.097062
44	1	0	1.260056	0.773909	1.079968
45	6	0	-0.205321	2.630030	-1.987536
46	1	0	-1.357369	0.859249	-2.336082
47	6	0	0.770550	3.273736	-1.197606

48	1	0	2.046869	3.030311	0.534381
49	1	0	-0.589985	3.109818	-2.878236
50	7	0	-0.519602	-0.666837	-0.287719
51	6	0	2.918943	5.900384	-0.056656
52	6	0	1.904708	6.149875	0.899500
53	6	0	4.109282	6.655420	0.069615
54	6	0	2.071514	7.054322	1.928539
55	1	0	0.967296	5.606369	0.842657
56	6	0	4.282060	7.576544	1.081281
57	1	0	4.899115	6.551040	-0.662740
58	6	0	3.272662	7.801512	2.056077
59	1	0	5.201670	8.146390	1.105334
60	7	0	3.446340	8.708175	3.065587
61	6	0	-7.893378	-0.635596	-0.035265
62	6	0	-6.011434	-2.166487	-0.768214
63	6	0	-6.983848	-1.058536	-1.088940
64	6	0	-6.904639	-0.534880	-2.366524
65	6	0	-6.524364	-3.430420	-0.599718
66	6	0	-7.912864	-3.711032	-0.825982
67	6	0	-5.760996	-4.559605	-0.157747
68	6	0	-7.641482	0.599114	-2.830605
69	6	0	-6.024754	-1.104446	-3.342394
70	7	0	-8.209151	1.528744	-3.242857
71	7	0	-5.312793	-1.556060	-4.145616
72	7	0	-9.032413	-3.972074	-1.005805
73	7	0	-5.188072	-5.497026	0.226335
74	6	0	1.281563	4.615366	-1.503938
75	6	0	0.552679	5.614373	-2.105374
76	6	0	2.704459	4.926094	-1.114711
77	6	0	3.693669	4.232181	-1.788681
78	6	0	1.150297	6.866872	-2.467725
79	6	0	-0.847510	5.525281	-2.396157
80	7	0	1.605778	7.890535	-2.781712
81	7	0	-1.989178	5.507567	-2.621744
82	6	0	3.381450	3.368958	-2.887507
83	6	0	5.081966	4.265177	-1.447108
84	7	0	3.150178	2.658144	-3.780454
85	7	0	6.217101	4.237090	-1.187393
86	6	0	3.531188	-4.374578	1.291905
87	6	0	4.420711	-4.926523	0.204668
88	6	0	3.789342	-4.754924	2.585133
89	6	0	3.833270	-5.868582	-0.621043
90	6	0	3.111363	-4.218763	3.728928
91	6	0	4.440949	-6.431630	-1.785792
92	6	0	2.507784	-6.342927	-0.359298
93	7	0	2.602451	-3.789664	4.683441
94	7	0	4.892143	-6.903317	-2.750383
95	7	0	1.429354	-6.731220	-0.153208
96	6	0	4.809125	-5.714941	2.896073
97	7	0	5.626780	-6.491729	3.181573
98	1	0	-8.109190	-0.740687	3.370958
99	1	0	1.263801	7.190415	2.635813

100	1	0	7.551883	-1.663881	1.098963
101	6	0	4.676436	9.485883	3.152099
102	1	0	5.554388	8.835436	3.238511
103	1	0	4.812396	10.135040	2.277675
104	1	0	4.637988	10.116681	4.039574
105	6	0	2.368337	8.971104	4.010342
106	1	0	1.470287	9.351815	3.507656
107	1	0	2.093823	8.067765	4.568491
108	1	0	2.697730	9.721429	4.728252
109	6	0	9.966850	-1.474219	0.459309
110	1	0	11.004349	-1.213150	0.253674
111	1	0	9.332008	-0.672921	0.062475
112	1	0	9.835187	-1.512444	1.547990
113	6	0	10.718754	-3.475962	-0.868567
114	1	0	10.947307	-4.434990	-0.386867
115	1	0	10.444559	-3.671015	-1.911996
116	1	0	11.625307	-2.871764	-0.866268
117	6	0	-11.722795	1.214836	2.823962
118	1	0	-11.565467	2.140264	2.257982
119	1	0	-12.400281	0.570386	2.249181
120	1	0	-12.215235	1.474138	3.760676
121	6	0	-10.129106	0.221648	4.498137
122	1	0	-10.060323	-0.862519	4.653717
123	1	0	-9.177362	0.673824	4.802825
124	1	0	-10.908834	0.608362	5.153517

Total Energy (HF) = -3416.0786516 Hartrees
